# Luminescence reveals variations in local structural order of calcium carbonate polymorphs formed by different mechanisms

**DOI:** 10.1038/s41598-019-52587-7

**Published:** 2019-11-07

**Authors:** Michael B. Toffolo, Giulia Ricci, Luisa Caneve, Ifat Kaplan-Ashiri

**Affiliations:** 1 Institut de Recherche sur les Archéomatériaux-Centre de Recherche en Physique Appliquée à l’Archéologie (IRAMAT-CRP2A), UMR 5060 CNRS, Université Bordeaux Montaigne, 8 Esplanade des Antilles, Pessac, 33607 France; 2 Dipartimento di Geoscienze, Università degli Studi di Padova, Via Giovanni Gradenigo 6, Padova, 35131 Italy; 3 ENEA, Technical Unit for the Development of Applications of Radiations, CR Frascati, Via Enrico Fermi 45, Frascati, 00044 Italy; 4 Department of Chemical Research Support, Weizmann Institute of Science, 234 Herzl Street, Rehovot, 7610001 Israel

**Keywords:** Solid-state chemistry, Infrared spectroscopy, Mass spectrometry, Analytical chemistry, Inorganic chemistry, Materials chemistry, Physical chemistry, Excited states, Chemical engineering, Structural materials, Applied physics, Chemistry, Engineering, Materials science, Physics, Optics and photonics, Applied optics, Lasers, LEDs and light sources

## Abstract

In nature, calcium carbonate (CaCO_3_) in the form of calcite and aragonite nucleates through different pathways including geogenic and biogenic processes. It may also occur as pyrogenic lime plaster and laboratory-precipitated crystals. All of these formation processes are conducive to different degrees of local structural order in CaCO_3_ crystals, with the pyrogenic and precipitated forms being the least ordered. These variations affect the manner in which crystals interact with electromagnetic radiation, and thus formation processes may be tracked using methods such as X-ray diffraction and infrared spectroscopy. Here we show that defects in the crystal structure of CaCO_3_ may be detected by looking at the luminescence of crystals. Using cathodoluminescence by scanning electron microscopy (SEM-CL) and laser-induced fluorescence (LIF), it is possible to discern different polymorphs and their mechanism of formation. We were thus able to determine that pyrogenic calcite and aragonite exhibit blue luminescence due to the incorporation of distortions in the crystal lattice caused by heat and rapid precipitation, in agreement with infrared spectroscopy assessments of local structural order. These results provide the first detailed reference database of SEM-CL and LIF spectra of CaCO_3_ standards, and find application in the characterization of optical, archaeological and construction materials.

## Introduction

Calcium carbonate (CaCO_3_) is a relatively common mineral on the Earth’s crust and occurs mainly in the forms of calcite and aragonite. Calcite, the stable polymorph at ambient conditions, is present mostly in geologic contexts, as it is the main component of limestone, chalk, travertine, marble and it is often found in unconsolidated sediments^1^. Aragonite, one of the metastable polymorphs, is predominantly found in biologic contexts, being formed by several organisms and in particular by mollusks^2^. Both polymorphs may be produced by humans through pyrotechnological processes, in the form of ash and lime plaster^3,4^, and by precipitation^5,6^. Considered their widespread occurrence, the crystallographic characterization of calcite and aragonite holds considerable value for a large number of research fields that require accurate knowledge of the formation path and preservation state of these minerals, including geology, geochemistry, environmental science, biomineralization, cultural heritage, construction engineering and industrial chemistry^1,2,5,7–9^. One of the properties of CaCO_3_ that can be used to assess the different formation processes of calcite and aragonite is its degree of crystallinity, the latter broadly defined as perfect order in three dimensions at the atomic level^10^. In this study, we refer to crystallinity in terms of crystallite size and defects in crystal structure.

Geogenic, biogenic and pyrogenic formation processes affect crystal properties such as habit, domain size and density, and thus are conducive to different degrees of local structural order, or crystallinity, in calcite and aragonite. For instance, single geogenic crystals nucleate from supersaturated solutions and grow over a long time, producing well-defined crystal faces as a result of three-dimensional periodic order over macroscopic distances^1^. Several biogenic crystals nucleate from an amorphous calcium carbonate (ACC) precursor crystallized by organisms^11^, and may show different degrees of order within the same individual based on their anatomical location and function^12^. Pyrogenic crystals form through the thermal decomposition of CaCO_3_ into CaO (quicklime), which readily reacts with water and CO_2_ in air to form again CaCO_3_ in a rapid process that leads to the incorporation of impurities and lattice distortions in relatively small crystallites^13,14^. These variations in atomic order affect the manner in which different types of calcite and aragonite interact with electromagnetic radiation, and thus formation processes may be tracked by using analytical methods that address long- and short-range atomic order. X-ray diffraction (XRD) is the reference method for assessing structural differences in crystals at the medium and long range^10^. Short-range order is better detected at a molecular level using Raman spectroscopy and Fourier transform infrared spectroscopy^15^ (FTIR). This is the case of poorly ordered phases, such as ACC and pyrogenic carbonates^4,16^. In addition, FTIR can distinguish between crystals formed by different mechanisms using the so-called “grinding curve” method, which exploits variations in peak broadening and intensity caused by distinct densities of structural defects^17^. Grinding curves allow detecting subtle differences in atomic order within heterogeneous materials that are of importance in biomineralization, materials science and archaeology^12,18–20^.

Structural defects that cause changes in diffraction patterns and infrared spectra may be identified in calcite and aragonite also by observing their luminescence. When irradiated with a high-energy electron beam, non-localized valence electrons in insulator and semi-conductor solids are temporarily promoted to an excited state. Upon decay to ground state, some electrons are trapped in lattice defects that occupy discrete energy levels. These are referred to as luminescence centers. Generally, two types of centers may be distinguished. Intrinsic centers are defects in the crystal structure, such as anion vacancies or lattice distortions and involve band-to-band recombination of electron and hole pairs. Extrinsic centers correspond to ion substitutions. The luminescence of carbonate minerals depends mainly on the abundance of Mn^2+^ activators, whereas the presence of Fe^2+^ has a fundamental role as quencher due to its capability in suppressing luminescence^21^. More specifically, the different types of luminescence in CaCO_3_ are caused by: i) substitutions of Mn^2+^ for Ca^2+^; ii) substitutions of rare earth elements (REE) for Ca^2+^; iii) dislocations of the [CO_3_]^2–^ moiety; iv) oxygen vacancies and broken Ca-O bonds^22–24^. When electrons vacate these traps, photons are emitted in the visible spectrum with wavelengths that are diagnostic for specific minerals^25^. This phenomenon, called cathodoluminescence (CL), has been studied to understand CaCO_3_ formation and diagenesis in geogenic and biogenic systems^22^. Similarly, electrons excited to a higher energy level by a laser beam with sufficient energy to activate allowed electronic transitions may generate fluorescence radiation in the UV and visible ranges; this is called laser-induced fluorescence (LIF) and finds application in the characterization of organic and inorganic molecules, as well as geogenic forms of calcite. It has been used as a diagnostic tool in biology and art with successful results thanks to its capability to perform remote, non-destructive and non-invasive qualitative analyses^26–28^. Luminescence techniques are used in order to investigate and interpret the composition and structure of organic molecules and minerals and find application in several fields of medicine, biology, geoscience, materials science and industry. Carbonates, as well as silicates and phosphates, are largely studied since they occur in sedimentary and igneous rocks and play an important role as industrial products and biomaterials^29^.

Despite the large amount of luminescence data available for calcite and aragonite formed in geogenic and biogenic systems, little is known about their pyrogenic counterpart. To date, pyrogenic CaCO_3_ has been investigated only in archaeological studies that focused on lime plaster affected by diagenesis, which do not provide information on the crystallinity of the original lime binder^22,30,31^. In addition, all of the previous CL studies on archaeological lime plaster and most studies on geogenic and biogenic CaCO_3_ have been conducted with a hot cathode attached to a petrographic microscope, which do not allow high-resolution characterization (i.e. <1 µm) and are based on subjective color interpretation rather than wavelength measurement, ultimately leading to discrepancies in the assessment of structural defects and crystallinity. Furthermore, the paucity of LIF studies of carbonate materials and especially pyrogenic carbonates requires a systematic characterization approach of standard reference samples. It thus follows that a comparison of LIF and CL analyses carried out on the same reference collection may provide essential complementary information. Therefore, here we report the first study of CL and LIF in experimental, aggregate-free lime plasters, thermally altered mollusk shells, and precipitated crystals composed of calcite and aragonite. In order to compare these materials with a known standard, we adopted a systematic approach that included the analysis of several geogenic and biogenic reference materials for both calcite and aragonite. Using high-resolution cathodoluminescence performed via field-emission scanning electron microscopy (SEM-CL), LIF and inductively coupled plasma mass spectrometry (ICP-MS), we show that pyrogenic and precipitated specimens are characterized by a strong blue luminescence, as opposed to the dominant orange luminescence of the starting material. We also demonstrate a correlation between luminescence wavelength and local structural order of CaCO_3_ crystals determined with FTIR. These results provide the first reference database for both SEM-CL and LIF of calcite and aragonite, and find application in the structural characterization of modern binders used in bioarchitecture and cement research, in the preservation of cultural heritage, and in industrial chemistry.

## Results and Discussion

All of the reference standard materials (Table 1) match the expected composition based on FTIR analysis except for Calcite 2, which was originally thought to be aragonite, and Marble 5, which is composed mainly of dolomite instead of calcite. Some of the lime plasters include traces of calcium hydroxide or minor CaCO_3_ polymorphs (either calcite or aragonite; see Supplementary Information and Supplementary Fig. S1). Elemental compositions obtained with ICP-MS are shown in Supplementary Table S2.

**Table 1:** List of calcite and aragonite specimens analyzed with SEM-CL. The asterisk (*) marks specimens analyzed also using LIF and ICP-MS.

Sample	Formation process	Provenience	Phase identification by FTIR
Calcite 1	Geogenic (spar)	Chihuahua, Mexico	Calcite
Calcite 2*	Geogenic (aragonite spar recrystallized to calcite)	Chihuahua, Mexico	Calcite
Calcite 3*	Geogenic (spar)	Chihuahua, Mexico	Calcite
Calcite 4*	Geogenic (spar)	Unknown locality, USA	Calcite
Calcite 5	Geogenic (spar)	Mato Grosso, Brazil	Calcite
Chalk 1*	Geogenic (sedimentary)	Nesher Ramla, Israel	Calcite
Chalk 2	Geogenic (sedimentary)	Megiddo, Israel	Calcite, silica
Limestone 1*	Geogenic (sedimentary)	Sde Boker, Israel	Calcite
Limestone 2*	Geogenic (sedimentary)	Sde Boker, Israel	Calcite
Marble 1*	Geogenic (metamorphic)	Carrara, Italy	Calcite
Marble 2*	Geogenic (metamorphic)	Carrara, Italy	Calcite
Marble 3*	Geogenic (metamorphic)	Carrara, Italy	Calcite
Marble 4*	Geogenic (metamorphic)	Paros, Greece	Calcite
Marble 5*	Geogenic (metamorphic)	Mount Pentelikon, Greece	Dolomite, traces of calcite
Nari	Geogenic (recrystallized chalk)	Tell es-Safi/Gath, Israel	Calcite
Shell C1	Pyrogenic (*Glycymeris* *insubrica* heated to 400 °C)	Ashkelon, Israel	Calcite
Shell C2	Pyrogenic (archaeological burnt *Glycymeris* *insubrica*)	Ashkelon, Israel	Calcite
Plaster C1*	Pyrogenic	Starting material: nari Tell es-Safi/Gath	Calcite, traces of calcium hydroxide
Plaster C2	Pyrogenic	Starting material: chalk Nesher Ramla	Calcite, traces of calcium hydroxide
Plaster C3	Pyrogenic	Starting material: chalk Nesher Ramla	Calcite, traces of aragonite
Plaster C4*	Pyrogenic	Starting material: chalk Nesher Ramla	Calcite, traces of aragonite
Aragonite 1*	Geogenic (spar)	Loma Badá, Spain	Aragonite
Aragonite 2	Geogenic (spar)	Molina de Aragón, Spain	Aragonite
Aragonite 3*	Geogenic (spar)	Minglanilla, Spain	Aragonite
Aragonite 4	Geogenic (spar)	Tazouta, Morocco	Aragonite
Aragonite 5	Geogenic (spar)	Toussit, Morocco	Aragonite
Lisan aragonite	Geogenic (evaporite)	Dead Sea, Israel	Aragonite
Shell A1*	Biogenic (*Glycymeris* *insubrica*)	Ashkelon, Israel	Aragonite
Kettle aragonite	Pyrogenic (scale from boiling water)	Rehovot, Israel	Aragonite
Plaster A1	Pyrogenic	Starting material: *Glycymeris* *insubrica* shells	Aragonite, traces of calcite and calcium hydroxide

### SEM-CL: calcite

**Figure 1:**
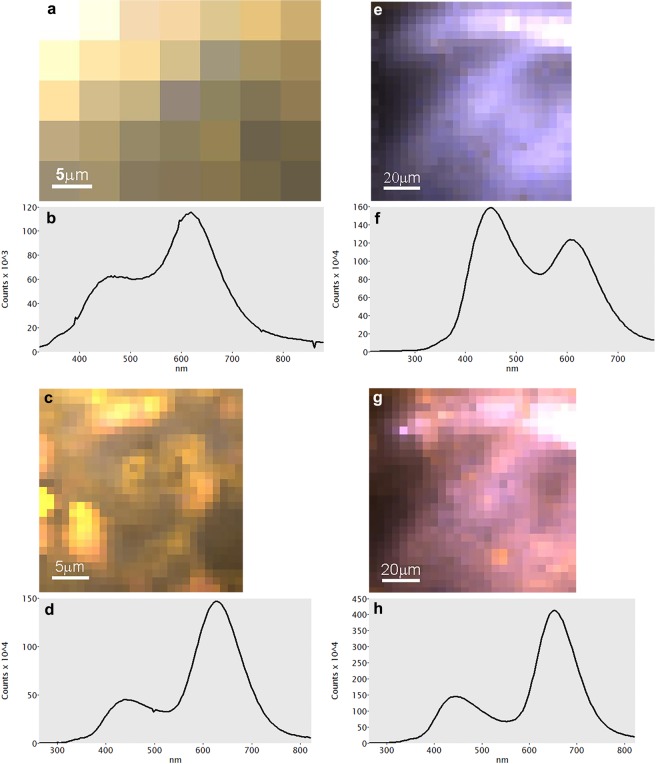
Representative SEM-CL spectra of geogenic calcites, including colored pixel map. (**a**,**b**) Limestone 1. (**c**,**d**) Marble 1. (**e**,**f**) Marble 4. (**g**,**h**) Marble 5.

**Figure 2:**
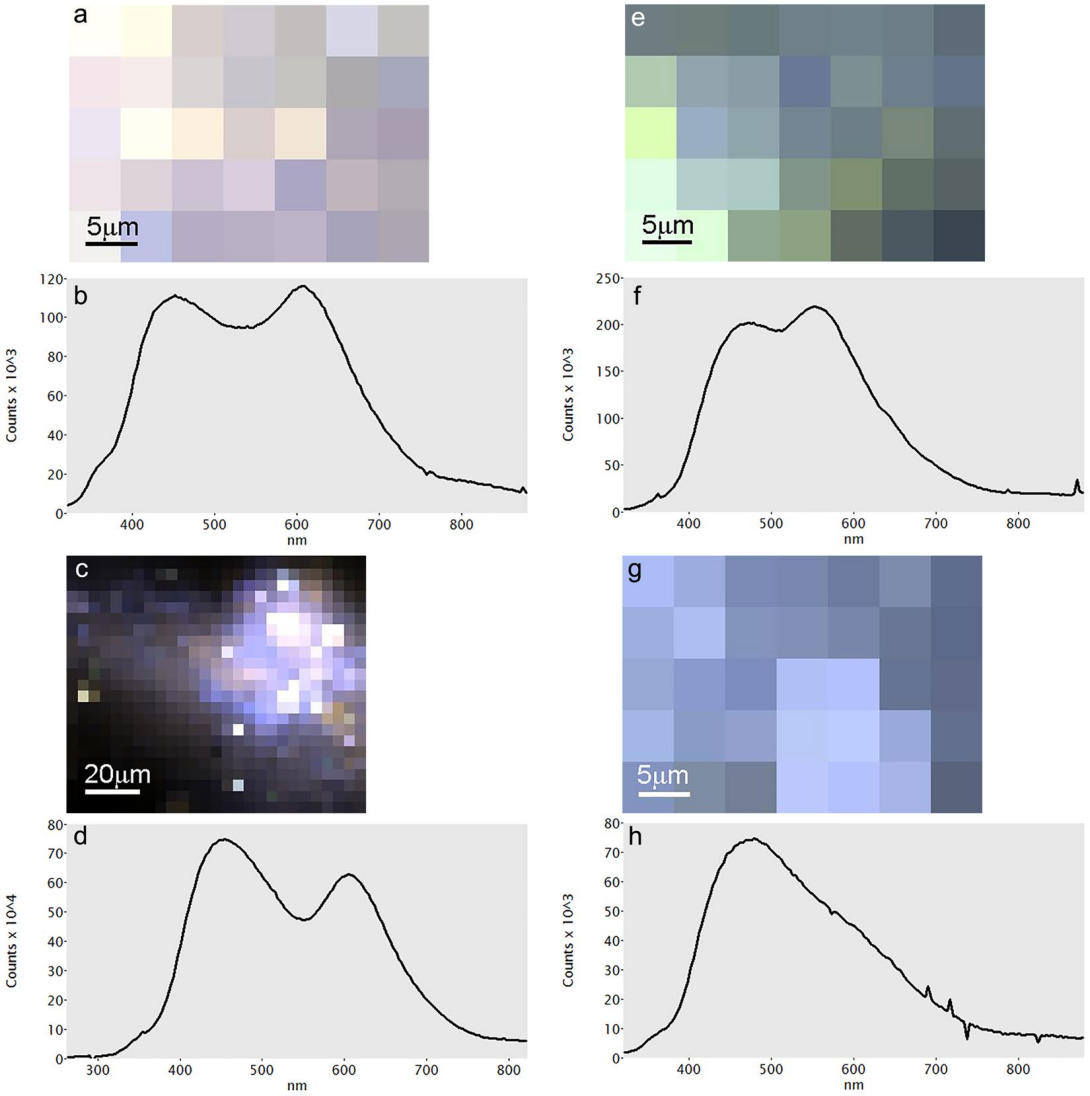
Representative SEM-CL spectra of pyrogenic samples with relative starting material, including colored pixel map. (**a**,**b**) Chalk 1. (**c**,**d**) Plaster C2. (**e**,**f**) Shell A1. (**g**,**h**) Plaster A1.

**Figure 3:**
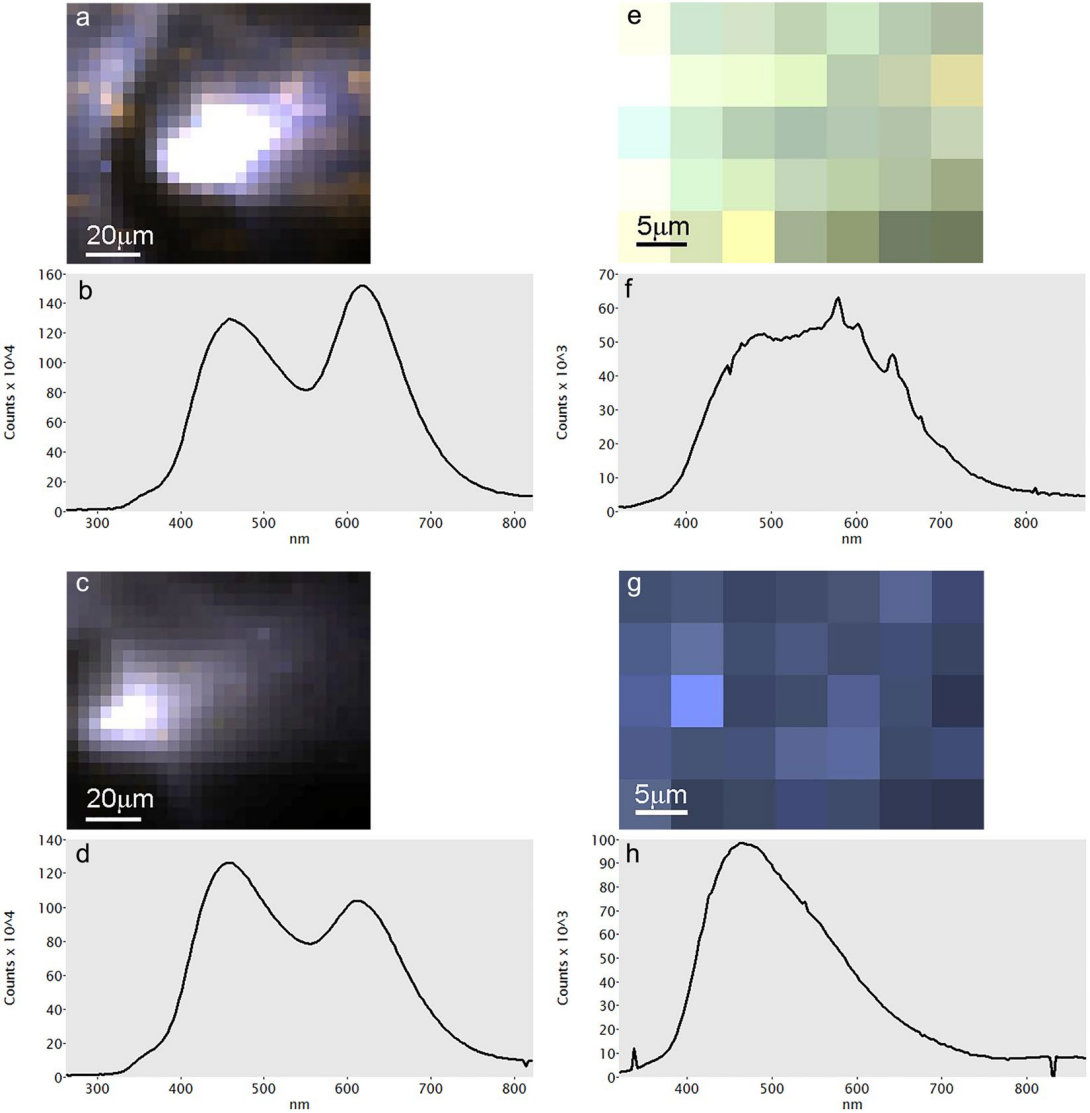
Representative SEM-CL spectra of geogenic and pyrogenic samples, including colored pixel map. (**a**,**b**) Shell C1. (**c**,**d**) Shell C2. (**e**,**f**) Lisan aragonite. (**g**,**h**) Kettle aragonite.

The results of SEM-CL measurements are presented in Supplementary Table S3, where the main emission bands for each sample together with their intensity and full width at half maximum (FWHM) are listed. Spectrum imaging maps with pixels and representative spectra are displayed in Figs 1–3 and Supplementary Figs S2–S6. All samples exhibit one emission band in the range 518–648 (±10) nm (green-red), and most of them also show a second emission band in the range 425–469 (±10) nm (violet-blue). The CL of calcite-based materials can be linked to two luminescence centers, namely Mn^2+^ substituting for Ca^2+^ (orange-red) and intrinsic lattice distortions (violet-blue)^25,32^. The same applies to aragonite-based materials, with the difference that Mn^2+^ substituting for Ca^2+^ generates green-yellow luminescence^33^.

The five calcite spar samples emit in the lower energy range 518–623 nm (green-orange) (Supplementary Fig. S2a–c), while only Calcite 1, 2 and 5 emit also in the higher energy range 441–457 nm (violet-blue). When comparing the intensity of the two bands for these three calcite samples, it can be observed that for Calcite 2 and 5 the higher energy band is more intense (opposite to Calcite 1) and narrower (according to the FWHM) compared to Calcite 1. The geogenic sedimentary samples (chalk and limestone) show two emission bands, with the lower energy in the range 613–658 nm (orange-red) and the higher energy in the range 425–469 nm (violet-blue). All four samples demonstrate higher intensity of the lower energy bands (Fig. 1a,b). In the case of marble samples there are two emission bands as well. The lower energy bands range between 605–646 nm (orange-red) and the higher energy bands occur around 436–443 nm (violet) (Fig. 1c,d). Looking at their intensities, only Marble 4 demonstrates higher intensity for the high-energy band opposite to all other marble samples (Fig. 1e,f). The Marble 5 low-energy band is shifted towards higher wavelengths compared to other marbles due to Mn^2+^ substituting for Mg^2+^, as the specimen is composed of dolomite (Fig. 1g,h). Nari exhibits two emission bands at 607 nm (orange) and 444 nm (violet). One major trend common to all of the geogenic specimens is the higher intensity of the orange emission compared to the blue one. The latter was not observed in Calcite 3 and 4, which are extremely rich in Mn as indicated by ICP-MS results (Supplementary Table S2). This trend is in agreement with previous studies of geogenic carbonate rocks^21,23,25,34,35^. One notable exception is Marble 4, which exhibits a dominant blue luminescence. This could be attributed to a lack of Mn^2+^ and abundance of Fe^2+^ in the original limestone, although the occurrence of orange emissions in most of the acquired spectra may suggest that metamorphic processes acting on limestone caused a number of distortions in the calcite crystal lattice, which gave rise to intrinsic luminescence centers. The same process may have affected other marble samples that present blue emissions. At this stage, the exact cause of the shallow blue intrinsic emission in geogenic carbonates is unknown, although it has been attributed to Ca^+^ and CO_3_^-^ centers created by radiation damage^34,36^.

Pyrogenic samples exhibit some variability in their emission bands. Plaster samples emit at wider ranges, 592–615 nm (orange) and 440–464 nm (violet-blue) for the lower and higher energy bands, respectively. Plaster C1 and C2 show higher intensity for the low energy bands compared to the higher energy bands and Plaster 3 and 4 show the opposite. Remarkably, Plaster C2 shows a stronger blue emission compared to its starting material, Chalk 1 (Fig. 2a–d). The burnt shells have similar emission bands at 616, 611 (orange), 460 and 454 nm (blue; the band pass is 20 nm), but their intensity for each band is opposite when compared (Fig. 3a–d). Therefore, pyrogenic calcite samples show an opposite trend compared to geogenic ones, i.e. the blue emission is stronger than the orange emission. The cause of the latter should be attributed to the occurrence of Mn^2+^ in Ca^2+^ sites as in geogenic samples, whereas the blue band may be a composite of different energy levels, including lattice distortions that generate intrinsic luminescence centers. This is indicated by residuals from peak fitting (Supplementary Fig. S7), which shows that the main blue emission masks other minor emissions at specific energy levels. These may represent other structural defects that are currently unknown, especially distortions caused by the rapid nucleation of nm-sized CaCO_3_ crystals out of Ca(OH)_2_^4^. Considering that the starting materials used to obtain Plasters C1, C2, C3 and C4 contain notable amounts of Mn^2+^ as indicated by their dominant orange emissions (Supplementary Table S3), it is interesting to note that only shallow orange emissions appear in the final product (Fig. 2). MnCO_3_ sites in the starting material are lost due to the decomposition of carbonate groups at the elevated temperatures necessary to obtain CaO. FTIR analysis of the latter excluded the presence of any type of carbonate absorption^4^. Therefore, we postulate that when CaO is hydrated with water to produce Ca(OH)_2_, the resulting high Eh (>1) favors the precipitation of Mn in oxide and hydroxide forms (non-luminescent), rather than carbonate^30,35^. In addition, it should be taken into account that the rate of precipitation of CaCO_3_ is much higher compared to MnCO_3_^37^. Moreover, Mn may change valence when exposed to high temperatures, after the breakdown of carbonate groups, and thus be unsuitable for substitution of Ca^2+^ during plaster carbonation^38^. All of these factors together may contribute to the overall lack of Mn^2+^ substitutions in lime plaster compared to chalk and limestone. A process similar to plaster production has been observed by Kusano *et al*.^39^ in geogenic skarn formations, where limestone and dolomite are altered to CaO by contact with hydrothermal fluids and show strong blue emissions together with minor orange ones. Shell C1 and C2, which were heated to relatively low temperature, also show strong blue emissions (Fig. 3), whereas the pristine aragonitic *Glycymeris* shell exhibits a dominant green band (Fig. 2). In this case, rather than Mn displacement due to carbonate breakdown, the cause of the blue emission may be sought in the transition from calcite to aragonite. The latter is an example of reconstructive polymorphism, whereby atomic bonds are broken and structural units are reassembled into different structures^40^. This process requires a considerable amount of energy, in this case represented by heat, which can promote the occurrence of defects in the newly formed calcite crystals. Defects may thus constitute intrinsic centers that generate blue luminescence.

### SEM-CL: aragonite

The geogenic and biogenic aragonite standards analyzed herein align with previous CL studies, as indicated by their dominant green-yellow emissions^25,33^. Aragonite spar and Lisan samples yielded a significant number of spectra characterized by multiple sharp peaks, evidence of their REE content (Supplementary Table S2 and S3). Peaks at 603–606 and 643–648 nm may be associated with Sm^3+^ and Pr^3+^/Tb^3+^, respectively^34^, and are especially abundant in Aragonite 3. Some of the spectra collected for these samples exhibit only two emissions, similar to calcite specimens, and therefore were selected as representative of luminescence centers not caused by REE. The lower energy bands range between 619–648 nm (orange-red), whereas for higher energy the band changes from sample to sample (Supplementary Fig. S2d–f). For instance, Aragonite 1 has no higher-energy band and Aragonites 2 and 3 exhibit higher-energy bands at 603 and 606 nm (orange). Aragonite 4 and 5 and Lisan aragonite have higher-energy bands in the range 441–481 nm (violet-blue) (Fig. 3e,f). Looking at the intensities of the different bands and different samples only Aragonite 5 shows higher intensity for the higher-energy band. Shell A1 has two bands at 544 and 447 nm (green and violet, respectively) (Fig. 2e,f).

The two pyrogenic aragonite samples, Plaster A1 (Fig. 2g,h) and Kettle aragonite (Fig. 3g,h) have close emission bands at 535, 531 (green), 453 and 449 nm (blue). Some of the spectra collected for Plaster A1 show the traces of REE, which are represented by several sharp peaks overlapped onto the blue and orange emission bands. Plaster A1 shows only blue emissions, as opposed to its starting material Shell A1 (Figure 2e–h). Remarkably, also pyrogenic aragonite samples show a dominant blue emission as observed for the pyrogenic calcite ones (Figs 2 and 3). More specifically, Plaster A1 is the result of the breakdown of carbonate groups at high temperatures and subsequent displacement of Mn^2+^, which precipitates in oxide and hydroxide form^35^. Kettle aragonite instead precipitates at lower temperature out of boiling water, and involves the rapid precipitation of needle-shaped crystals that are likely characterized by a high density of structural defects^18^, hence the blue luminescence.

### LIF: calcite

**Figure 4:**
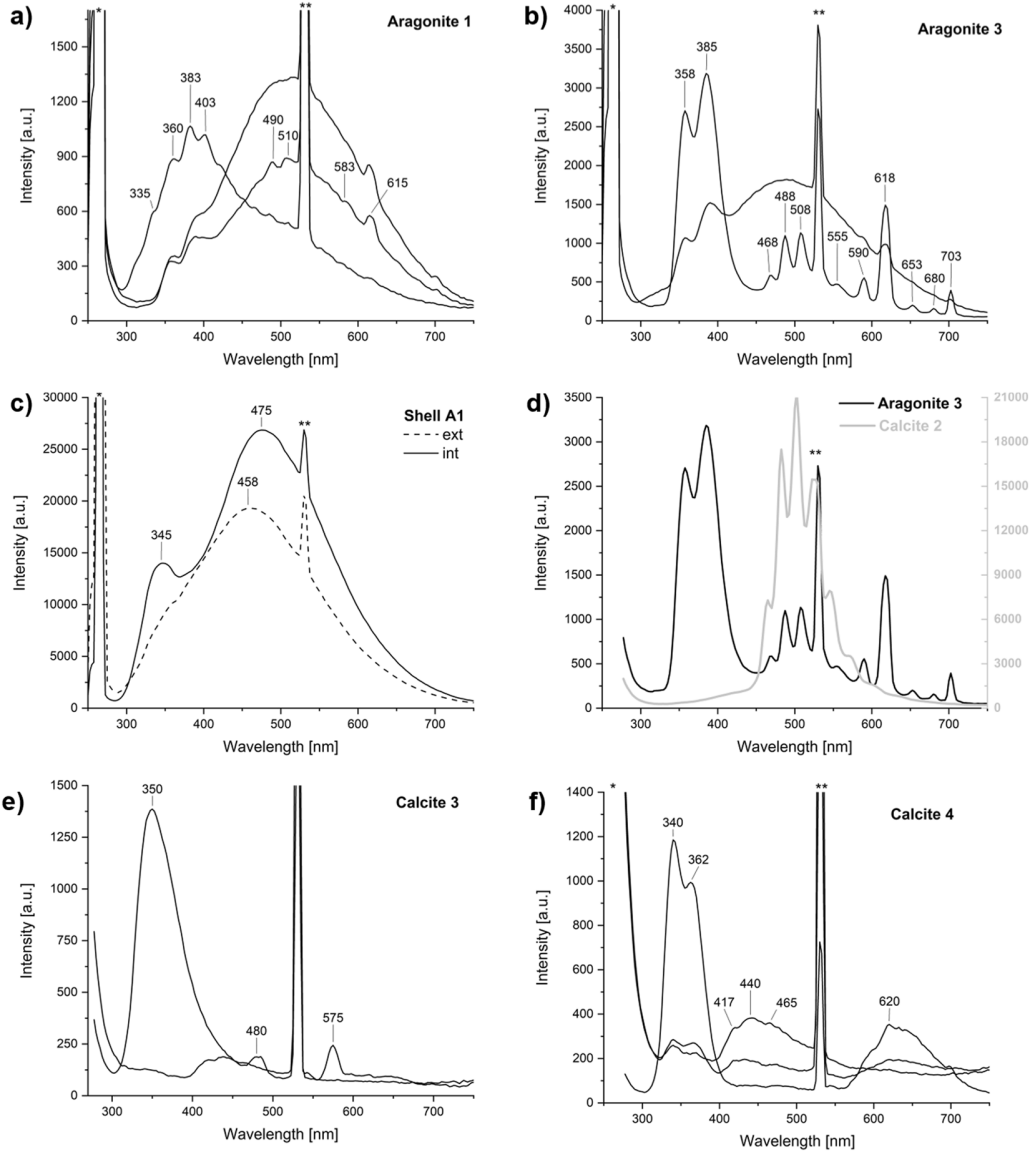
Representative LIF spectra of geogenic and biogenic samples of calcite and aragonite. For each sample, 16 spectra were acquired by scanning point by point and covering a surface of ~1.5 cm^2^. Characteristic and/or medium spectra for each sample are here presented. Some samples present heterogenous composition of the analyzed surface and more than one spectrum of the same sample is shown: Aragonite 1 (**a**); Aragonite 3 (**b**); external and internal surface of Shell A1 (**c**); Calcite 2 compared with Aragonite 3 (**d**); Calcite 3 (**e**); Calcite 4 (**f**).

**Figure 5:**
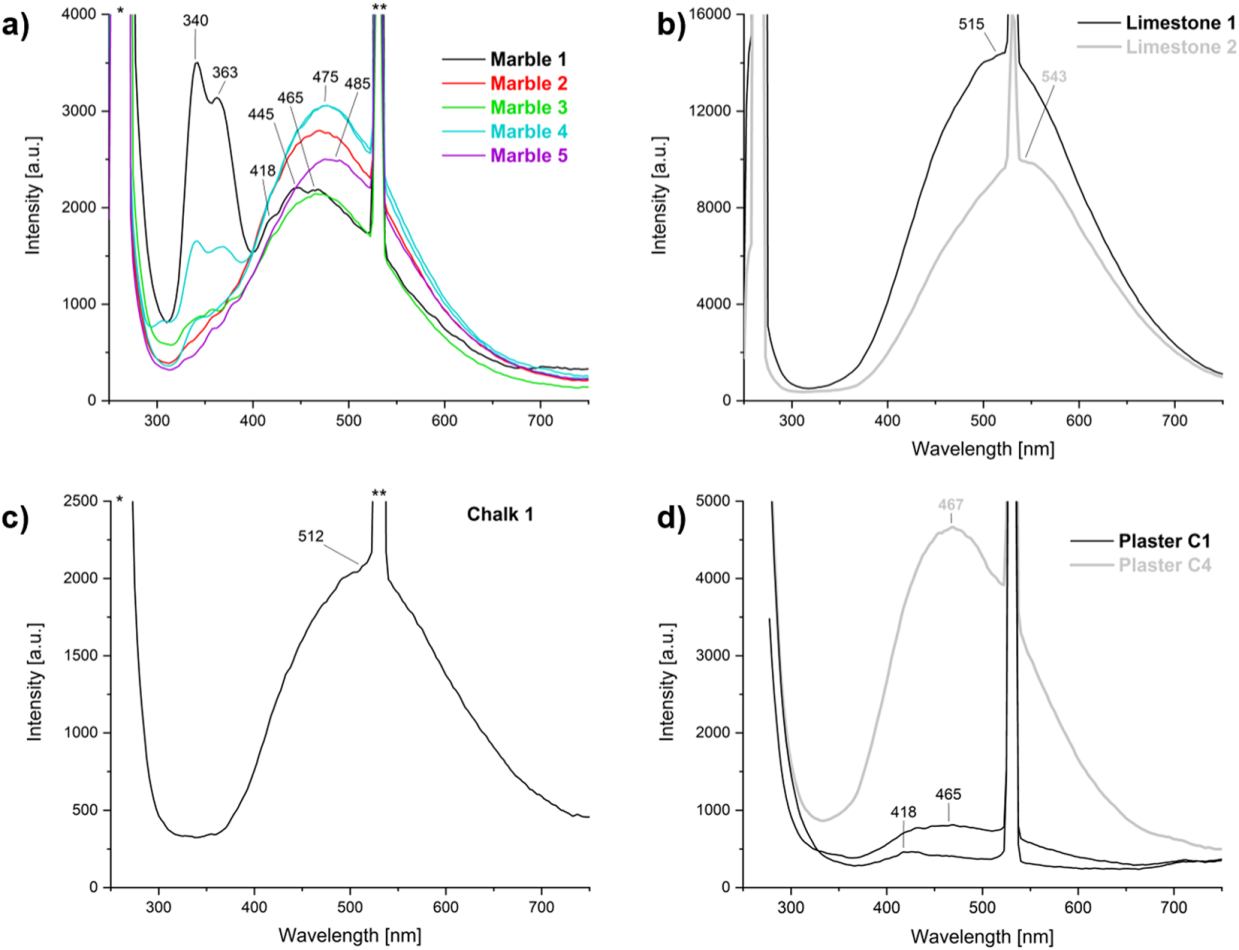
Representative LIF spectra of geogenic and pyrogenic samples of calcite. For each sample, 16 spectra were acquired by scanning point by point and covering a surface of ~1.5 cm^2^. Characteristic and medium spectra for each sample are here presented: all of the marble samples analyzed in this study (**a**); Limestone 1 and 2 (**b**); Chalk 1 (**c**); Plaster C1 and C4 (**d**).

Representative spectra and main detected peaks are shown in Figs 4 and 5 and in Supplementary Table S3, respectively. Calcite spar specimens show various fluorescence bands in the range 250–750 nm due to the presence of activator elements as well as lattice distortions. Calcite 2 stands out for its strong, narrow peaks between 450 and 600 nm (blue-orange), caused by uranyl ions that act as activators, as shown by U concentrations in the ICP-MS results (Supplementary Table S2). These results are in agreement with the genesis of the Calcite 2 sample, which is aragonite spar recrystallized into calcite. Calcite 3 presents a broad weak band in the range 400–500 nm (violet-blue), probably due to Pb^2+^ substitutions in the crystal lattice^41^ or to the recombination of electrons of (Ca^+^-CO_3_^-^)-centers for the intrinsic emission band^21^. Furthermore, the weakness of the luminescence emission may be due to the high degree of purity of the sample, in agreement with the significantly low concentration of activator elements detected in this sample. Calcite 4 presents strong bands at 340 and 362 nm (near UV) that can be associated with the characteristic bands of Pb^2+^ and Ce^3+^ I, and Ce^3+^ II. The broad band between 415 and 500 nm (violet-blue) is probably due to the intrinsic emission of electron-hole recombination where a CO_3_^−^ ion plays the role of the hole and Ca^+^ the role of the electron^21,36^. This band may be also related to induced radiation and/or other elements such as Tm^3+^ and Dy^3+ ^^42^. A distinct band at 620 nm, observed in most of the Calcite 4 spectra, is caused by Mn^2+^ substituting for Ca^2+^ in the CaCO_3_ lattice and representing the transition between the ^4^T_1g_ and ^6^A_1g_ electronic states of Mn^2+ ^^43^ (Fig. 4). Marble and limestone samples show some similarities with calcite spar (Fig. 5). Transitions at 340–363 nm and 470 nm are more pronounced in Marble 1 and 4 and may be due to structural defects with possible contribution of Ce^3+^, Pb^2+^, Tm^3+^ and Dy^3+^. Marble 5 shows the main luminescence band slightly shifted to lower energy probably because it is comprised mostly of dolomite. The band at 470 nm is not symmetrical and thus it is comprised of at least two distinct components. Therefore, peak deconvolution was carried out to identify specific peaks within broad asymmetric bands. As displayed in Table 2 and Supplementary Fig. S8, marble samples exhibit a band at 570–604 nm that can be associated with Mn^2+^ and Mn^4+^ substituting for Ca^2+^. These results seem to be consistent with previous studies on marbles^43,44^. The spectra of Chalk 1 and Limestone 1 and 2 are characterized by a strong and broad band around 515–540 nm. Peak deconvolution suggests that the main band may be composed of three different contributions at 436–449 nm, 509–541 nm and 620–660 nm. These are caused, respectively, by induced radiation and intrinsic emission, by Tm^3+^, Tb^3+^ and Dy^3+^ substitutions, and by Mn^2+^-Mn^4+^ as main activator centers. Furthermore, the presence of Fe^2+^ as an efficient luminescence quencher, has to be considered since it can suppress luminescence at 400–420 nm and bands at 520 nm up to 700 nm were observed^21^.

**Table 2:** Peak positions (in nm) calculated by fitting LIF peaks of marble, limestone and chalk samples (see Supplementary Fig. S8).

Samples	Peak 1	Peak 2	Peak 3	Peak 4	Peak 5
Marble 1	340	364	464	531	604
Marble 2	—	—	454	531	531
Marble 3	346	—	471	531	577
Limestone 1	439	518	625	531	—
Limestone 2	449	541	660	531	—
Chalk 1	436	509	620	531	—

Analyses on pyrogenic calcite Plaster C1 and C4 provide the first systematic LIF characterization of pyrogenic calcite. Plaster C1 exhibits weak bands between 400–470 nm, whereas Plaster C4 presents a strong and broad band centered at 467 nm (Fig. 5). Previous studies demonstrate that lime binder or burnt limestone produced from a luminescent starting material show dull luminescence due to the Mn^2+^/Fe^2+^ ratio in the calcite linked to the changing of Eh and pH conditions in setting mortars^30,34^. In this study, we show with SEM-CL that experimental plasters are characterized by a strong blue component at ~450 nm, which is not dependent on pH, but rather on the displacement of Mn after the breakdown of carbonate groups. As suggested above, it appears that the paucity of MnCO_3_ sites in plaster compared to the starting material may be linked to the elevated Eh of Ca(OH)_2_, to the low rate of precipitation of MnCO_3_ as opposed to CaCO_3_, and possibly to changes in Mn valence upon heating at high temperature. It thus seems that LIF results are consistent with SEM-CL.

### LIF: aragonite

Aragonite is highly luminescent and more than one luminescence transition is possible^45^ (Fig. 4). The LIF results presented herein are in agreement with existing literature and include the characteristic green-yellow emission (500–600 nm) caused by Mn^2+^ substitution for Ca^2+^ in the orthorhombic structure of aragonite^45–47^. This emission can be linked to the transition from an unsplit and excited state ^4^T_1g_ to the ground state ^6^A_1g_^25,47,48^. Multiple peaks in the range 300–450 nm of samples Aragonite 1 and 3 may be associated with intrinsic structural defects as well as with the presence of non-bridging oxygen hole centers (NBOHC) linked to the large concentration of defects at grain boundaries and cracks^47,49^. The orange emission around 620 nm may be due to Mn^4+^ and/or octahedrally coordinated Mn^2+^. Indeed, different parameters should be taken into account when considering the contribution of Mn in aragonite, such as orbital transition state, valence state, lattice position, and the coordination of Mn^50^. Furthermore, other elements may also act as activators in aragonite. In the collected spectra of Aragonite 1 and 3, some of the localities analyzed present characteristic peaks in the range 450–580 nm comparable to those caused by the occurrence of U(VI) in aragonite, as indicated also by the ICP-MS results (Supplementary Table S2). As reported in the literature, uranyl substitutes in the CaCO_3_ structure and it is preferentially incorporated into aragonite than calcite because aragonite provides a more stable coordination promoting uranyl long-term retention until it converts to calcite^46,51^. Luminescence in carbonate shells, as reported for Shell A1, is caused by the same physical phenomena as those observed in geogenic CaCO_3_, i.e. Mn^2+^ substitution for Ca^2+^ and structural defects. Shell A1 exhibits strong luminescence in the 400–500 nm range, which is consistent with the occurrence of aragonite; the presence of a band at 345 nm in the inner surface can be associated with an intrinsic emission center.

### Comparison of SEM-CL and LIF

In this study we provide the first reference database of combined SEM-CL and LIF of CaCO_3_ standard materials, including experimental, aggregate-free lime plasters composed of pyrogenic calcite and aragonite (Supplementary Table S3). To the best of our knowledge, the luminescence behavior of the latter has never been investigated before. Comparing SEM-CL and LIF results, the latter include bands at 400–500 nm that are probably due to the nature of the excitation source. In fact, it has been demonstrated that blue bands are more prominent under short-wave UV excitation^41^. Emission intensities, on the other hand, are dependent on the energy of the excitation method, activator, sensitizer and quencher concentrations. Interpretation of fluorescence spectra is a known challenge due to the characteristically broad signals and the lack of a systematic investigation of the fluorescence properties of materials, especially if induced by a UV laser. However, it appears that LIF and SEM-CL can provide complementary information, with the former more effective at lower wavelengths of the visible spectrum and near-UV, and the latter more sensitive to higher wavelengths and near-IR. For instance, blue emissions in Calcite 3 and 4 were detected with LIF, but not with SEM-CL. In addition, LIF seems to yield more accurate results with regard to some REE, especially U(VI), Pb^2+^, Ce^3+^ and Tm^3+^. LIF and SEM-CL may thus be used in combination to assess the wavelength of the main emission in both calcite and aragonite.

### Structural order in CaCO_3_

**Figure 6:**
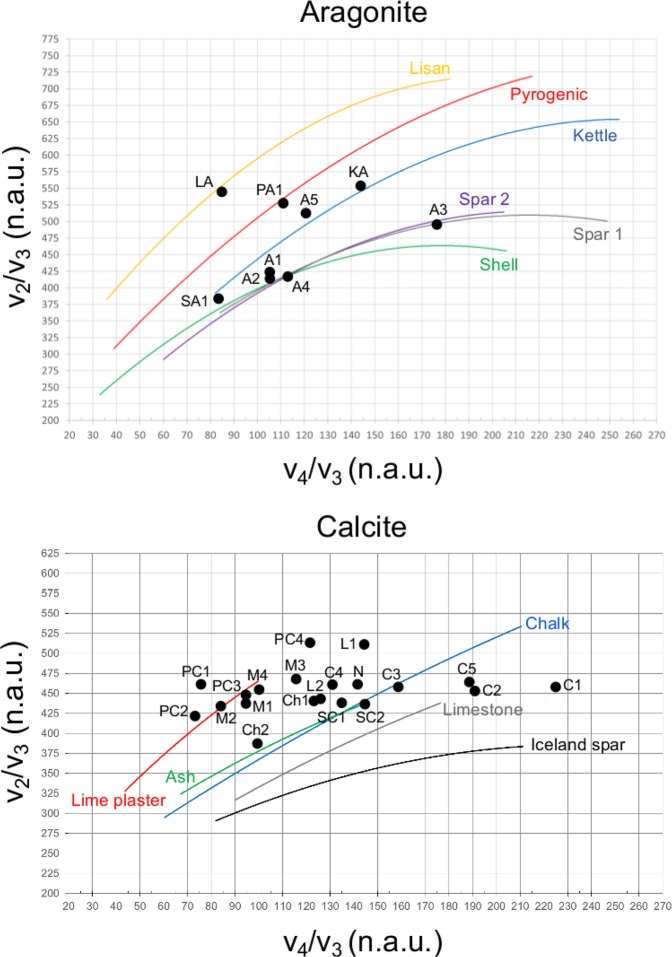
FTIR grinding curves of calcite and aragonite, showing the location of samples analyzed in this study. The ν_2_ and ν_4_ intensities are normalized to the intensity of the ν_3_ absorption and multiplied by 1000 for convenience (n.a.u.: normalized absorbance units). Normalized intensities are listed in Supplementary Table S1. A1: Aragonite 1. A2: Aragonite 2. A3: Aragonite 3. A4: Aragonite 4. A5: Aragonite 5. LA: Lisan aragonite. KA: SA1: Shell aragonite. Kettle aragonite. PA1: Plaster aragonite 1. C1: Calcite 1. C2: Calcite 2. C3: Calcite 3. C4: Calcite 4. C5: Calcite 5. Ch1: Chalk 1. Ch2: Chalk 2. L1: Limestone 1. L2: Limestone 2. M1: Marble 1. M2: Marble 2. M3: Marble 3. M4: Marble 4. N: Nari. SC1: Shell calcite 1. SC2: Shell calcite 2. PC1: Plaster calcite 1. PC2: Plaster calcite 2. PC3: Plaster calcite 3. PC4: Plaster calcite 4.

The normalized ν_2_ and ν_4_ infrared intensity of each calcite and aragonite reference material was plotted in the relative grinding curve charts, which highlighted a significant pattern in the luminescence wavelength of the specimens (Fig. 6). By comparing their degree of local structural order with SEM-CL and LIF spectra, it appears that all specimens characterized by poor atomic order consistently exhibit blue luminescence, in several cases as the dominant band (Figs 2 and 5). This is especially apparent for pyrogenic calcite and aragonite produced through a CaO step^13,14^. As stated above, the shallow orange emissions are presumably caused by the displacement of Mn^2+^ out of the crystal lattice of pyrogenic CaCO_3_ upon its thermal decomposition. As a result, only blue luminescence from intrinsic centers is visible, and its high intensity is somewhat in contrast with previous reports of shallow and broad intrinsic luminescence in CaCO_3_ poor in Mn ions, as is the case of cave speleothems^34^ and mineralized tissues of marine organisms^33^. Here we show with SEM-CL and LIF that pyrogenic CaCO_3_ includes a strong band at ~450 nm. We propose that its components, located at slightly different energy levels, represent defects in the crystal lattice of calcite and aragonite caused by the rapid carbonation of Ca(OH)_2_ derived from thermally produced CaO. The CL of Shell C1, C2 and Kettle aragonite lend support to this hypothesis. Shells were heated to 400 °C, which is well below the minimum temperature required to convert CaCO_3_ into CaO (~650 °C)^4^. However, the rearrangement of aragonite crystals that turn into calcite involves the incorporation of defects caused by heat, and this results in a strong blue emission besides the orange emission generated by Mn^2+^, which is not displaced from the calcite crystal structure (Fig. 3). This is confirmed by FTIR grinding curves (Fig. 6). Kettle aragonite precipitates instantly out of boiling water, and that too determines distortions in the crystal lattice (Fig. 6) that give rise to blue luminescence. Among the poorly ordered geogenic samples, Lisan aragonite (evaporitic precipitate) is comprised of a major blue emission and sharp shoulder peaks in the green caused by REE, in agreement with FTIR results (Fig. 6). Marble 1, 2 and 4 are poorly ordered based on FTIR and include strong blue emissions, which could be related to the elevated temperatures and pressures that acted on limestone during metamorphic processes.

### Applications

Given the correlation with FTIR grinding curves, SEM-CL and LIF could be used to predict the degree of atomic order of calcite and aragonite in pyrogenic samples, and therefore their state of preservation. In particular, the fields of cultural heritage conservation and archaeology may benefit from a thorough analysis of the luminescence of lime-based products. Historic lime plasters and lime binders in mortars will show dominant blue emissions if the pyrogenic CaCO_3_ fraction is pristine. Instead, when the intact pyrogenic binder is exposed to H_2_CO_3_ from groundwater, it tends to dissolve and enter solution as Ca(HCO_3_)_2_, which then precipitates again as CaCO_3_ upon evaporation of water^52^. Following Ostwald’s Rule of Stages^53^, secondary CaCO_3_ nucleates as larger crystals, more ordered at the atomic level compared to the pyrogenic precursor. In addition, the dissolution of pyrogenic CaCO_3_ in acidic conditions favors the mobilization of Mn ions that occur in form of oxides in the lime binder, whereas the precipitation of secondary CaCO_3_ favors the incorporation of environmental Mn present in groundwater. The resulting crystals will thus show the characteristic orange emission caused by Mn^2+^. This model has been well studied in the layered structure of cave stalagmites, which typically exhibit faint blue luminescence but often include layers with bright orange luminescence caused by the presence of detrital Mn from the external enviroment^54^. Similarly, CaCO_3_ mollusk shells devoid of Mn in their pristine form have been shown to incorporate environmental Mn upon diagenesis, which results in orange CL emissions^55^. Therefore, CL and LIF could be used as proxies to assess the state of preservation of lime mortars and plasters in historic architectures and artworks, including restoration materials^56^. LIF would be particularly effective given its ability to collect data without harming the integrity of the sample. Furthermore, SEM-CL and LIF may be used in the screening of archaeological lime binders selected for radiocarbon (^14^C) dating. Previous studies performed using CL by optical microscopy highlighted the occurrence of “brown” to “purple” luminescence in lime binders^30,31^, which can be interpreted as the contribution of blue and orange emissions, similar to what has been observed here in experimental plasters. Recent advances in this field have shown that the integrity of pyrogenic CaCO_3_ crystals formed during plaster/mortar carbonation, i.e. the time of the reaction between Ca(OH)_2_ and atmospheric CO_2_, is crucial for the preservation of the original ^14^C signature necessary to obtain accurate age determinations^52,57,58^. Thus, SEM-CL and LIF can help discard unsuitable samples.

In addition to past lime binders, SEM-CL and LIF could make significant contributions to the structural characterization of modern binders used in bioarchitecture and cement industry. Properties such as solubility, which is different between aragonite and calcite^1^ and can affect the durability of binders, and post-depositional processes, like nucleation of secondary calcite and aragonite in cement^59^, may be tracked using luminescence^60^. The identification of pure aragonite produced starting from Ca(OH)_2_ holds considerable value also for the paper industry, where it is preferred over calcite as paper stock filler since it provides enhanced brightness and strength^61^. Moreover, trace-element and impurity fluctuations in the ppm range, often not detectable by common analytical methods such as X-ray fluorescence and SEM-EDS (energy dispersive x-ray spectroscopy), influence the luminescence emission in ultra-pure materials^32^. Therefore, alterations, new phases and the distribution of trace elements can be successfully revealed by surface-mapping heterogeneous carbonate materials through SEM-CL and LIF. This becomes especially important in the case of REE-doped synthetic CaCO_3_ crystals, which are preferentially produced through the carbonation of Ca(OH)_2_ under different settings, and are used in multifunctional applications such as solar cells, sensing and detection, and biological imaging^62–64^. For instance, binary CaCO_3_-Tb complexes used in the production of highly fluorescent composite materials could be readily investigated using both LIF and SEM-CL, due to the prominent Tb^3+^ emissions in both calcite and aragonite and the suppressed Mn^2+^ signal in pyrogenic carbonates^63^. Finally, it should be noted that the nucleation process of calcite and aragonite used in the development of biomimetic materials comprised of a combination of CaCO_3_ and biopolymers (e.g. chitin, cellulose) requires an extremely detailed characterization of crystal composition and properties, including lattice distortions, in order to control adhesive bonds between carbonate and polymer layer^5^. Considering their inherent advantages, luminescence techniques may thus be developed into standard analytical methods for the investigation and characterization of technical products such as building materials, biomaterials, and new optical materials^29,32^.

## Conclusions

The structural characterization of CaCO_3_ polymorphs formed by different mechanisms is of great importance for several research fields. In particular, CaCO_3_ obtained through pyrogenic and precipitation processes is used in a number of applications, including the study and conservation of cultural heritage, the characterization of binders, and the production of biomimetic and optical materials. The assessment of the degree of local structural order in CaCO_3_ crystals performed using benchmark methods such as XRD and FTIR may be complemented by SEM-CL and LIF. These techniques aim at characterizing the degree of purity of crystals through the detection of trace elements in both natural and synthetic materials. In this study we provided the first detailed comparison of SEM-CL and LIF spectra of CaCO_3_ and showed that pyrogenic calcite and aragonite exhibit mainly blue luminescence caused by heat-induced lattice distortions, as opposed to their starting materials. This is in agreement with FTIR and ICP-MS results. Therefore, this information may be used in the assessment of the degree of preservation of CaCO_3_ materials and in the preparation of doped synthetic crystals. Analyses may be carried out in a non-destructive way using LIF. Future work will investigate the possibility of quantifying the degree of local structural order in CaCO_3_ crystals.

## Materials and Methods

### Standard reference materials

Calcite and aragonite specimens formed through different mechanisms, and thus characterized by different degrees of atomic order, were selected for this study (Table 1). In particular, we focused on geogenic/biogenic and pyrogenic materials as examples of well-ordered and poorly ordered carbonates, respectively. Calcite 2 and 3 and aragonite 5 were purchased from Ward’s Science (Rochester, NY, USA), whereas aragonite 1, 2 and 4 were purchased from Mineralogical Research Company (San Jose, CA, USA). All other geogenic and biogenic samples were acquired during previous fieldwork projects or kindly provided by colleagues. In addition, we included calcite derived from the recrystallization of aragonite caused by exposure to elevated temperatures. A recent specimen of *Glycymeris* *insubrica* shell (C1) was collected on the Mediterranean shore at Ashkelon (Israel) and heated to 400 °C for 2 h in an electric muffle oven in air atmosphere to turn aragonite into calcite. An archaeological *Glycymeris* *insubrica* shell (C2) from the Bronze Age levels of ancient Ashkelon (~1400 BCE)^65^ was found in a combustion feature and turned out to be composed of calcite as a result of heating. Plaster C1 was prepared by heating nari powder to 900 °C for 6 h in an electric muffle oven in air atmosphere and by mixing the resulting CaO (quicklime) with excess deionized water; the plaster was left to carbonate at room conditions. Plasters C2, C3 and C4 were prepared by burning chalk cobbles in an open fire at temperatures between 600 and 900 °C for 8 h. Quicklime was hydrated with excess water and plasters were left to carbonate at room conditions. Plaster A1 was prepared by heating powdered *Glycymeris* *insubrica* shells to 900 °C for 12 h in an electric muffle oven in air atmosphere. Quicklime was left to carbonate at room conditions. Carbonation settings (relative humidity, CO_2_ partial pressure, temperature) were not monitored. At the time of this study, calcite plasters were 10 years old, whereas the aragonite plaster was 7 years old.

### Fourier transform infrared spectroscopy (FTIR)

The composition of all the samples analyzed in this study was determined using FTIR spectroscopy. A few milligrams of each standard material were homogenized and powdered in an agate mortar and pestle. About 0.1 mg were left in the mortar and mixed with approximately 0.5 mg of KBr (FTIR grade, Sigma-Aldrich), and pressed into a 7-mm pellet using a hand press (PIKE Technologies). Infrared spectra were obtained at 4 cm^−1^ resolution in 32 scans within the 4000–400 cm^−1^ spectral range using a Bruker Alpha spectrometer or a Thermo Fisher Scientific Nicolet iS5 spectrometer. Phase identification was performed using OMNIC v. 9, standard literature^66^ and the reference collection of FTIR spectra of standard materials provided by the Kimmel Center for Archaeological Science, Weizmann Institute of Science (http://www.weizmann.ac.il/kimmel-arch/infrared-spectra-library). The degree of atomic order of calcite and aragonite crystals was determined using the grinding curve methods of Regev *et al*.^19^ and Toffolo *et al*.^18^, which are based on the relation between the intensity of the ν_2_ and ν_4_ absorptions of calcite/aragonite, normalized to the intensity of the relative ν_3_ absorption. Macros Basic v. 8 was used to create a macro for the calculation of the normalized intensities and thus swiftly process a large number of spectra. The ν_2_/ν_3_ and ν_4_/ν_3_ ratios were multiplied by 1000 for convenience in creating grinding curve plots.

### Inductively coupled plasma mass spectrometry (ICP-MS)

Representative standard reference materials analyzed with both SEM-CL and LIF were investigated also using ICP-MS in order to obtain trace elements (Mn, Fe, U, Pb and REE) concentration related to the typical extrinsic luminescence centers present in calcite. ICP-MS measurements were carried out by a Thermo Scientific iCAP RQ ICP-MS system for ultratrace elemental analysis at the CNR-Institute for the Dynamics of Environmental Processes, located in the Department of Environmental Sciences at University Ca’ Foscari, Venice^67^. All the pre-analytical procedures were carried out in the clean laboratory available at the IGG-CNR-Institute, located in the Department of Geosciences at University of Padua. All materials used for sampling, treatment and storage of samples and solutions were carefully chosen, acid-cleaned and conditioned to minimize sample contamination^68^. Samples were ground and homogenized and 5 mg were digested in PFA vials with 3 ml of HNO_3_. The solutions were evaporated at 90 °C on an electric plate and the residues were dissolved with 10 ml of 5% v/v HNO_3_. High-purity water and reagents were used during sample preparation. A blank sample was also prepared. Element concentrations were calculated using an external calibration curve method and the calibration solutions were prepared in 5% v/v HNO_3_ by dilution from ICP multi-element standard solutions IMS-101, IMS-102 and IMS-104 (Ultra Scientific) at 11 different concentrations (0.01–500 ppb).

### Cathodoluminescence by scanning electron microscopy (SEM-CL)

Reference standard materials were powdered and about 5 mg of each sample were pressed into a 3-mm pellet using a hand press (PIKE Technologies). Pellets were mounted on SEM stubs using carbon tape and coated with a layer of carbon 7–8 nm thick, using carbon thread evaporation (Safematic CCU-010). To collect SEM-CL spectra we used a Gatan MonoCL4 Elite system equipped with a retractable diamond-turned mirror. The collected light first was imaged in panchromatic mode using a high-sensitivity photomultiplier tube with a spectral range of 160–930 nm. Then, the collected light was directed to a monochromator and a charge-coupled device for parallel spectroscopy. The spectral range was set to 300–800 nm with a band pass of 20 nm by choosing the 150 lines/mm grating centered on 550 nm and 1 mm entrance slit. The CL system is installed on a Zeiss Gemini SEM 500, a high-resolution SEM equipped with two-modes field emission gun. CL measurements were performed at 20 kV with an aperture of 20 µm in analytical gun mode (Supplementary Fig. S9). CL hypermaps were collected in spectrum imaging (SI) mode, the pixel size and exposure time were fixed for each sample according to its CL intensity. The simultaneous SEM images were collected using the SE2 detector (Everhardt-Thornely detector). For each sample the SI was collected at several different regions of interest (ROI), then the spectra were analyzed by Gaussian fitting in order to extract the emission wavelength, the intensity and the full width half maximum (FWHM) of the different bands (Supplementary Fig. S7).

### Laser-induced fluorescence (LIF)

LIF analyses were performed at the ENEA Research Centre in Frascati (Rome). Experiments were carried out using a radiation source Thomson DIVA diode pulsed Nd:YAG laser with excitation wavelength of 266 nm, a repetition rate of 20 Hz with a pulse duration of 8 ns and a laser fluence of 0.9 mJ/cm^2^. The LIF apparatus was designed and built at the ENEA laboratory^69,70^ and it is shown in Supplementary Fig. S10. The UV beam originating from the laser is reflected by a mirror at 45°, then crosses a perforated mirror (3 mm in diameter) and is directed towards the remote target through a mobile mirror. The latter is mounted on two motorized axial movements controlled by the computer. The collected fluorescence radiation is focused at the entrance of an optical fiber linked to a compact QE PRO spectrometer from Ocean Optics working in the range 200–900 nm. Appropriate filters were placed in front of the fiber in order to avoid the backscattered radiation and the second order of the Nd:YAG laser emission. No optical elements were used to collimate the laser beam and the resolution was approximately 1 to 2 mm, inferred from the spot size on the target. The spectrometer is connected to a PC where a LabView program allows the user to set experimental parameters, control the rotating mirror in order to perform the scanning of the sample’s surface, control data acquisition and conduct preliminary data analysis. Measurements were performed by scanning point by point and covering a surface of ca. 1.5 cm^2^. For each sample, 16 spectra were collected. Characteristic spectra were evaluated and spectral post-processing was carried out using Origin(Pro), Version 2018b, OriginLab Corporation, Northampton, MA, USA (Supplementary Fig. S8). Fresh breaks were investigated in order to avoid altered surfaces that may influence the luminescence signal^43^.

## Supplementary information

Supplementary Information
