# Supplementary material for: Luminescence reveals variations in local structural order of calcium carbonate polymorphs formed by different mechanisms
Source: Sci Rep. 2019 Nov 7;9:16170. doi: 10.1038/s41598-019-52587-7 (PMC6838102; doi:10.1038/s41598-019-52587-7)
Supplement: Supplementary file 1 — Supplementary Information [file 41598_2019_52587_MOESM1_ESM.pdf]

High-resolution cathodoluminescence and laser-induced fluorescence reveal variations in local structural order of calcite and aragonite formed by different mechanisms

Michael B. Toffolo[1], Giulia Ricci[2], Luisa Caneve[3], Ifat Kaplan-Ashiri[4]

[1]Institut de Recherche sur les Archéomatériaux-Centre de Recherche en Physique Appliquée à l'Archéologie (IRAMAT-CRP2A), UMR 5060 CNRS, Université Bordeaux Montaigne, 8 Esplanade des Antilles, Pessac 33607, France.

[2]Dipartimento di Geoscienze, Università degli Studi di Padova, Via Giovanni Gradenigo 6, Padova 35131, Italy.

[3]ENEA, Technical Unit for the Development of Applications of Radiations, CR Frascati, Via Enrico Fermi 45, Frascati 00044, Italy.

[4]Department of Chemical Research Support, Weizmann Institute of Science, 234 Herzl Street, Rehovot 7610001, Israel.

Supplementary Information

FTIR spectroscopy

In most cases, reference standard materials (Table 1) exhibit the diagnostic absorptions of the expected $CaCO_3$ polymorph indicated by the supplier, i.e. bands at 1430 (ν_3), 875 (ν_2) and 713 (ν_4) cm^{-1} for calcite and 1475 (ν_3), 1083 (ν_1), 858 (ν_2), 713 and 700 (ν_4) cm^{-1} for aragonite (Supplementary Figure S1). One notable exception is Calcite 2, which according to the supplier should be aragonite but exhibits peaks of calcite, presumably due to recrystallization caused by poor preservation conditions. In addition, Marble 2 contains a small amount of dolomite besides calcite, whereas Marble 5 is almost entirely made of dolomite, whose characteristic absorptions are located at 1448 (ν_3), 881 (ν_2) and 729 (ν_4) cm^{-1}. Among the experimental samples, Plaster C1 and C2 show the peaks of calcite and that of $Ca(OH)_2$ at 3644 cm^{-1} due to incomplete carbonation. Plaster A1 exhibits aragonite absorptions (with the ν_3 shifted to 1487 cm^{-1}) and shallow calcite peaks and shoulders besides the main aragonite component, whereas Plaster C3 and C4 include traces of aragonite represented by shallow peaks at 1083 and 858 cm^{-1}. The *Glycimeris* shell heated to 400 °C for 2 h is composed of calcite, as well as the burnt archaeological specimen.

The normalized ν_2 and ν_4 intensity of each calcite and aragonite reference material were plotted in the relative grinding curve charts (Supplementary Table S1 and Figure 6). Most specimens fall in the expected areas, in agreement with previously published results using similar materials[1,2]. Calcite 3 and 4 are slightly less ordered at the atomic level compared to other spar samples. Limestone 1 and 2 also appear to be closer to chalk in terms of local structural order. Marble 1-4, which are made of calcite, fall between the grinding curves of chalk and ash, thus showing a relatively low degree of order. Calcite in Shell C1 and C2 is characterized by a low degree of order, similar to calcitic ash.

ICP-MS

REE (Ce, Dy, Eu, Nd, Sm, Tb, Tm), Mn, Fe, Pb and U are the most common activator elements in carbonates, and their concentrations are listed in Supplementary Table S2. All of the samples show variable concentration of REE below 2 ppm. In particular, Ce concentration is higher in Calcite 4, Aragonite 3, Aragonite 1, Marble 1 and Limestone 2 (~30, 18, 4, 3 and 3 ppm respectively). U is mostly present in Aragonite 1 and Calcite 2, whereas Pb is present under 2 ppm except for Plaster C4, which contains ~3 ppm. Mn occurs in large concentrations in Calcite 4 and Calcite 3 with 2176 and 362 ppm, whereas in other samples it is under 20 ppm.

References

1 Regev, L., Poduska, K. M., Addadi, L., Weiner, S. & Boaretto, E. Distinguishing between calcites formed by different mechanisms using infrared spectrometry: archaeological applications. *Journal of Archaeological Science* **37**, 3022-3029 (2010).

2 Toffolo, M. B., Regev, L., Dubernet, S., Lefrais, Y. & Boaretto, E. FTIR-Based Crystallinity Assessment of Aragonite–Calcite Mixtures in Archaeological Lime Binders Altered by Diagenesis. *Minerals* **9**, 121 (2019).

Supplementary Figure S1

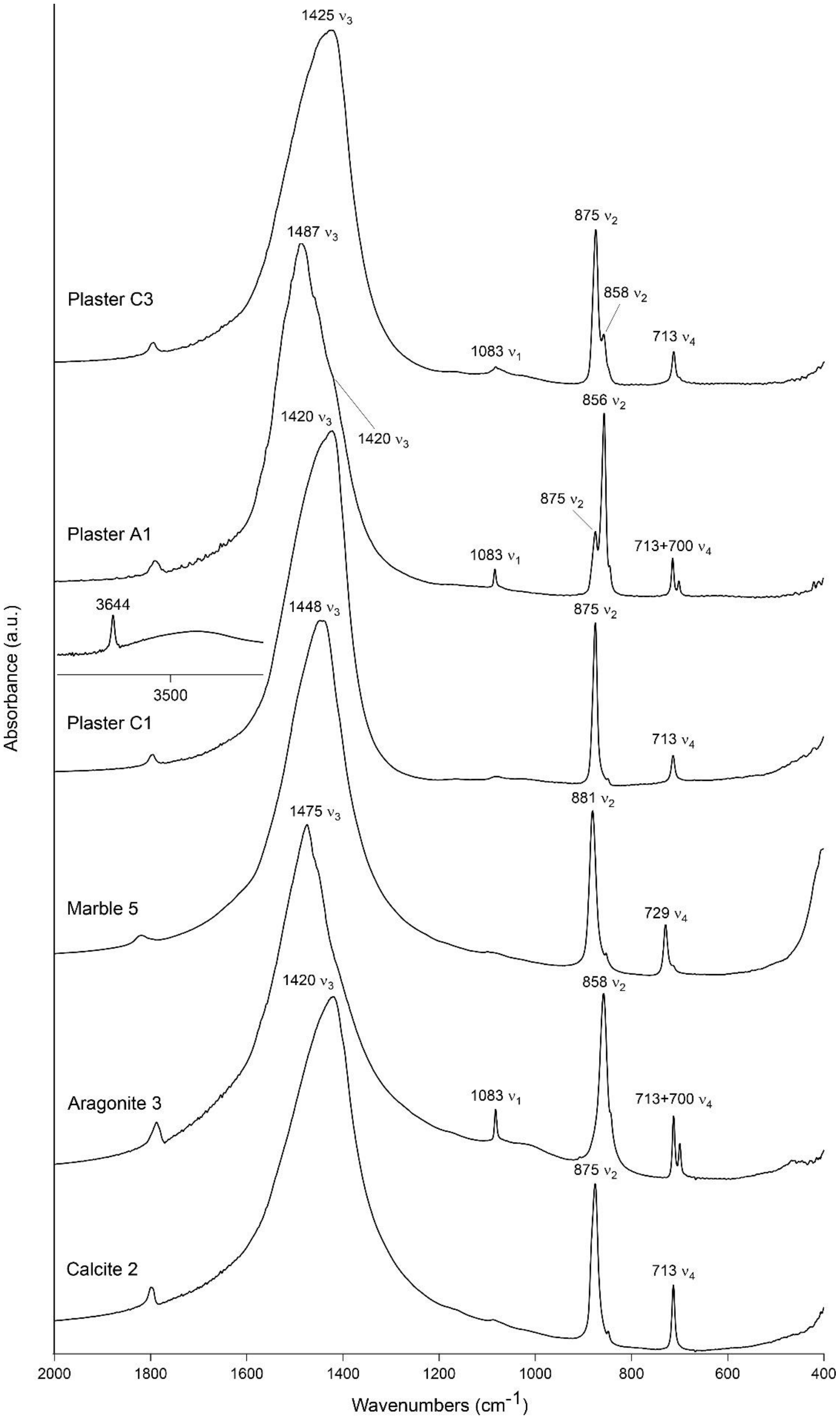

Supplementary Figure S1. Representative FTIR spectra of reference standard calcium carbonate materials analyzed in this study. The inset of Plaster C1 shows the location of the calcium hydroxide absorption band.

Supplementary Table S1. FTIR grinding curves results. Intensities of the ν_2 and ν_4 peaks of calcite and aragonite in normalized absorbance units (n.a.u.) for all the samples analyzed with SEM-CL (Table 1), including the full width at half maximum (FWHM) of the ν_3 absorption in wavenumbers (cm^{-1}). The method is not applicable (n/a) to Marble 5, which is composed of dolomite.

**Sample**	**ν_2 (n.a.u.)**	**ν_4 (n.a.u.)**	**ν_3 FWHM (cm^{-1})**
Calcite 1	463	224	179
Calcite 2	458	191	172
Calcite 3	456	148	167
Calcite 4	461	131	158
Calcite 5	462	188	175
Chalk 1	440	123	147
Chalk 2	386	99	119
Limestone 1	512	144	161
Limestone 2	438	126	140
Marble 1	441	94	143
Marble 2	430	84	153
Marble 3	470	116	157
Marble 4	454	100	158
Marble 5	n/a	n/a	n/a
Nari	466	142	145
Shell C1	438	135	155
Shell C2	428	143	146
Plaster C1	462	76	138
Plaster C2	423	73	144
Plaster C3	447	95	158
Plaster C4	511	122	168
Aragonite 1	425	106	106
Aragonite 2	415	106	98
Aragonite 3	493	178	164
Aragonite 4	414	113	99
Aragonite 5	519	121	149
Lisan aragonite	545	85	103
Shell A1	387	83	98
Kettle aragonite	552	143	159
Plaster A1	526	111	141

Supplementary Table S2. Trace element concentrations of selected samples obtained by ICP-MS.

Sample	Elements of interest for CL and LIF (ppm)										
	**Mn**	**Fe**	**Pb**	**U**	**Ce**	**Nd**	**Sm**	**Eu**	**Tb**	**Dy**	**Tm**
Calcite 2	0.959	121.904	0.782	3.595	0.054	0.031	0.007	0.002	0.002	0.004	0.003
Calcite 3	361.805	122.367	0.751	0.001	0.064	0.025	0.013	0.003	0.005	0.012	0.005
Calcite 4	2175.637	495.426	1.432	0.264	29.291	30.331	8.897	2.863	3.804	18.949	2.733
Chalk 1	5.212	270.753	0.559	0.413	0.564	0.265	0.070	0.041	0.038	0.058	0.032
Limestone 1	10.328	269.192	0.715	0.353	0.491	0.322	0.066	0.017	0.018	0.069	0.010
Limestone 2	12.074	1364.885	0.928	1.223	2.523	0.652	0.120	0.039	0.036	0.138	0.034
Marble 1	13.691	681.286	1.212	0.028	2.554	1.958	0.398	0.097	0.093	0.400	0.048
Marble 2	19.227	326.174	0.737	0.225	0.580	0.284	0.050	0.015	0.016	0.063	0.011
Marble 3	10.895	164.368	0.549	0.086	0.476	0.352	0.068	0.017	0.021	0.097	0.017
Marble 4	3.891	119.111	0.612	0.034	0.173	0.117	0.021	0.005	0.006	0.023	0.003
Marble 5	13.179	154.904	0.601	0.071	0.180	0.076	0.014	0.005	0.004	0.019	0.003
Plaster C1	17.822	1478.886	1.136	0.320	1.921	1.272	0.255	0.065	0.055	0.246	0.032
Plaster C4	13.505	835.392	2.982	0.906	1.116	0.503	0.105	0.024	0.017	0.066	0.007
Aragonite 1	16.929	1082.268	0.893	1.884	3.700	1.747	0.537	0.144	0.123	0.414	0.017
Aragonite 3	17.869	494.251	1.086	0.460	17.623	8.296	1.885	0.474	0.301	0.949	0.032
Shell A1	13.627	181.613	0.535	0.215	1.108	0.469	0.067	0.020	0.012	0.036	0.003

Supplementary Table S3. SEM-CL and LIF peak locations, and relative luminescence centers (a.u.: absorbance units; NBOHC: non-bridging oxygen hole centers).

Sample	CL bands (nm)	Intensity (a.u.)	FWHM (nm)	CL luminescence centers	LIF bands (nm)	LIF luminescence centers
Calcite 1	457	90774	109	Intrinsic	Not analyzed	-
	624	1071271	89	Mn^{2+}		
Calcite 2	441	421669	85	Intrinsic	465, 482, 502, 545, 572	U(VI)
	518	561974	186	Mn^{2+}	600	Mn^{2+}, Mn^{4+}
Calcite 3	619	2933294	76	Mn^{2+}	417, 440, 465	Ce^{3+}, recombination of electrons for intrinsic emission
Calcite 4	614	4781426	74	Mn^{2+}	340, 362	Pb^{2+}, Ce^{3+}
					417, 440, 465	Recombination of electrons for intrinsic emission, radiation induced, Tm^{3+} and Dy^{3+}
					620	Mn^{2+}
Calcite 5	449	718584	96	Intrinsic	Not analyzed	-
	551	532575	187	Mn^{2+}		
Chalk 1	425	110790	88	Intrinsic	542	Induced radiation, intrinsic emission, Tm^{3+}, Tb^{3+} and Dy^{3+}, Mn^{2+}
	613	153904	111	Mn^{2+}		
Chalk 2	469	831018	118	Intrinsic	Not analyzed	-
	625	2167417	106	Mn^{2+}		
Limestone 1	461	75094	130	Intrinsic	534	Induced radiation, intrinsic emission, Tm^{3+}, Tb^{3+} and Dy^{3+}, Mn^{2+}
	617	137251	119	Mn^{2+}		
Limestone 2	461	1242945	116	Intrinsic	546	Induced radiation, intrinsic emission, Tm^{3+}, Tb^{3+} and Dy^{3+}, Mn^{2+}
	658	1550492	102	Mn^{2+} (in Mg^{2+} position)		
Marble 1	441	324750	88	Intrinsic	340, 363	Pb^{2+}, Ce^{3+}

	621	1988034	91	Mn^{2+}	418, 445, 465	Recombination of electrons for intrinsic emission, radiation induced, Tm^{3+} and Dy^{3+}
Marble 2	443	745688	86	Intrinsic	470	Recombination of electrons for intrinsic emission, radiation induced, Tm^{3+} and Dy^{3+}
	612	2025013	90	Mn^{2+}		
Marble 3	440	402371	85	Intrinsic	470	Recombination of electrons for intrinsic emission, radiation induced, Tm^{3+} and Dy^{3+}
	610	985714	87	Mn^{2+}		
Marble 4	438	1428910	84	Intrinsic	340, 363	Pb^{2+}, Ce^{3+}
	605	1124080	107	Mn^{2+}	475	Recombination of electrons for intrinsic emission, radiation induced, Tm^{3+} and Dy^{3+}
Marble 5	436	1291146	84	Intrinsic	485	Recombination of electrons for intrinsic emission, radiation induced, Tm^{3+} and Dy^{3+}
	646	2183015	85	Mn^{2+} (in Mg^{2+} position)		
Nari	444	865159	85	Intrinsic	Not analyzed	-
	607	1324980	101	Mn^{2+}		
Shell C1	460	1307683	104	Intrinsic	Not analyzed	-
	616	5833153	121	Mn^{2+}		
Shell C2	454	1114286	100	Intrinsic	Not analyzed	-
	611	915759	135	Mn^{2+}		
Plaster C1	464	39810	131	Intrinsic	418	Radiation induced
	592	33466	160	Mn^{2+}	465	Intrinsic
Plaster C2	440	823744	94	Intrinsic	Not analyzed	-
	592	596315	128	Mn^{2+}		
Plaster C3	449	232627	93	Intrinsic	Not analyzed	-
	615	537628	92	Mn^{2+}		
Plaster C4	443	392137	89	Intrinsic	467	Intrinsic

	611	608599	101	Mn^{2+}		
Aragonite 1	648	22948	97	REE, Mn^{2+}	335, 360, 383, 402	Intrinsic, structural defects, NBOHC
					490, 510, 582	Mn^{2+}
					615	Mn^{2+}, Mn^{4+}
Aragonite 2	603, 644	2469560, 3359363	80, 39	REE, Mn^{2+}	Not analyzed	-
Aragonite 3	606, 643	1231384, 2011613	31, 48	REE, Mn^{2+}	358, 385	Structural defects, NBOHC
					468, 488, 510, 555, 590	Mn^{2+}, U(VI)
					618	Mn^{2+}, Mn^{4+}
Aragonite 4	465	39050	132	Intrinsic	Not analyzed	-
	628	411247	96	Mn^{2+}		
Aragonite 5	441	491757	78	Intrinsic	Not analyzed	-
	624	236449	110	Mn^{2+}		
Lisan aragonite	481	1943	132	Intrinsic	Not analyzed	-
	619	4666	106	Mn^{2+}		
Shell A1	447	115104	89	Intrinsic	345	Intrinsic, structural defects
	544	112736	122	Mn^{2+}	458, 475	Mn^{2+}
Kettle aragonite	453	46918	89	Intrinsic	Not analyzed	-
	535	42965	167	Mn^{2+}		
Plaster A1	449	38485	100	Intrinsic	Not analyzed	-
	531	34115	148	Mn^{2+}		

Supplementary Figure S2

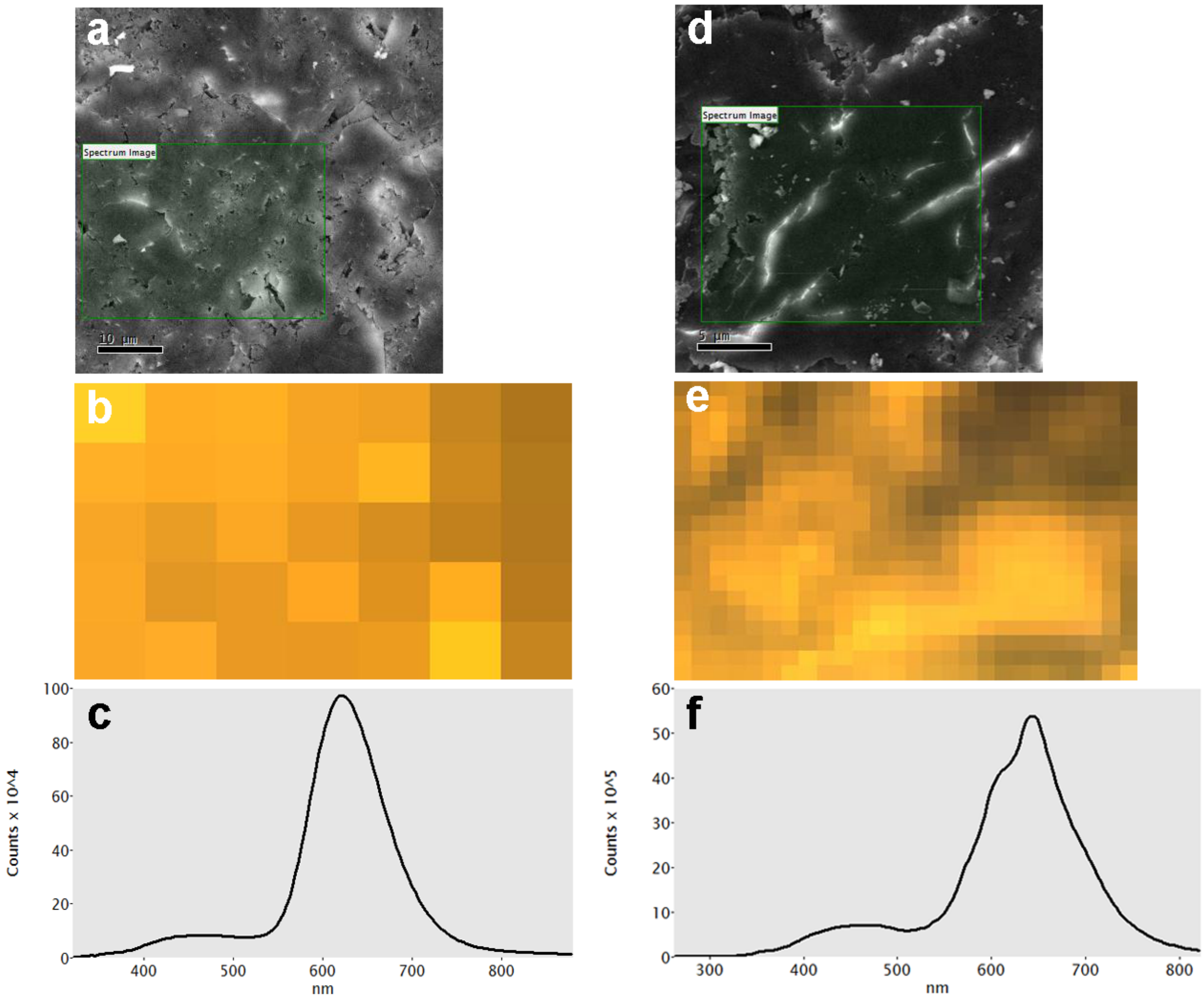

Supplementary Figure S2. SEM-CL spectra of calcite spar and aragonite spar. (**a**) SEM image of a region of interest in sample Calcite 1. (**b**) Spectrum imaging map of Calcite 1 showing emission colors; each pixel corresponds to a measurement. (**c**) Representative CL spectrum of Calcite 1. (**d**) SEM image of a region of interest in sample Aragonite 2. (**e**) Spectrum imaging map of Aragonite 2 showing emission colors; each pixel corresponds to a measurement. (**f**) Representative CL spectrum of Aragonite 2.

Supplementary Figure S3

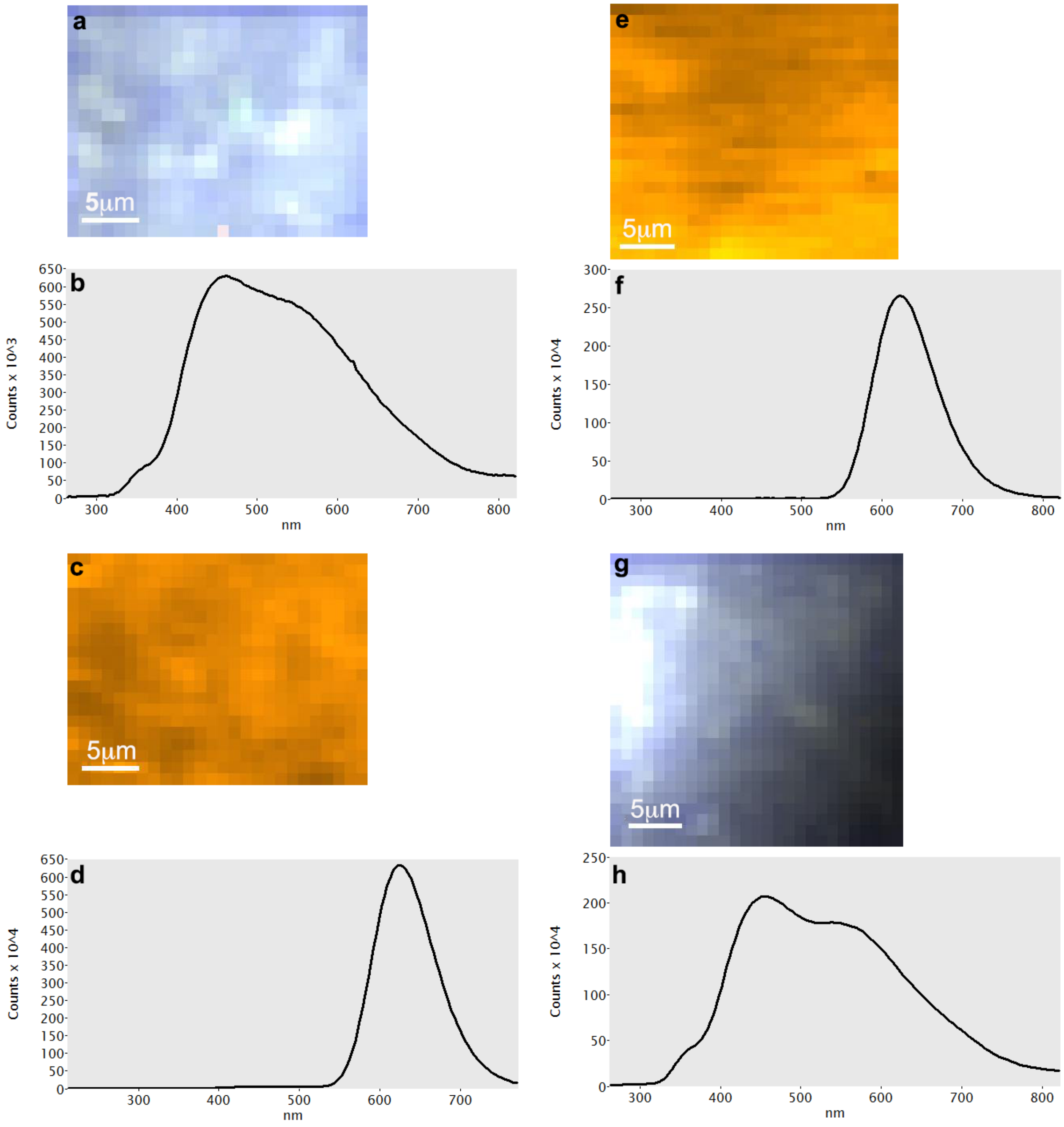

Supplementary Figure S3. Spectrum imaging maps and SEM-CL spectra of standard reference samples. (**a**, **b**) Calcite 2. (**c**, **d**) Calcite 3. (**e**, **f**) Calcite 4. (**g**, **h**) Calcite 5.

Supplementary Figure S4

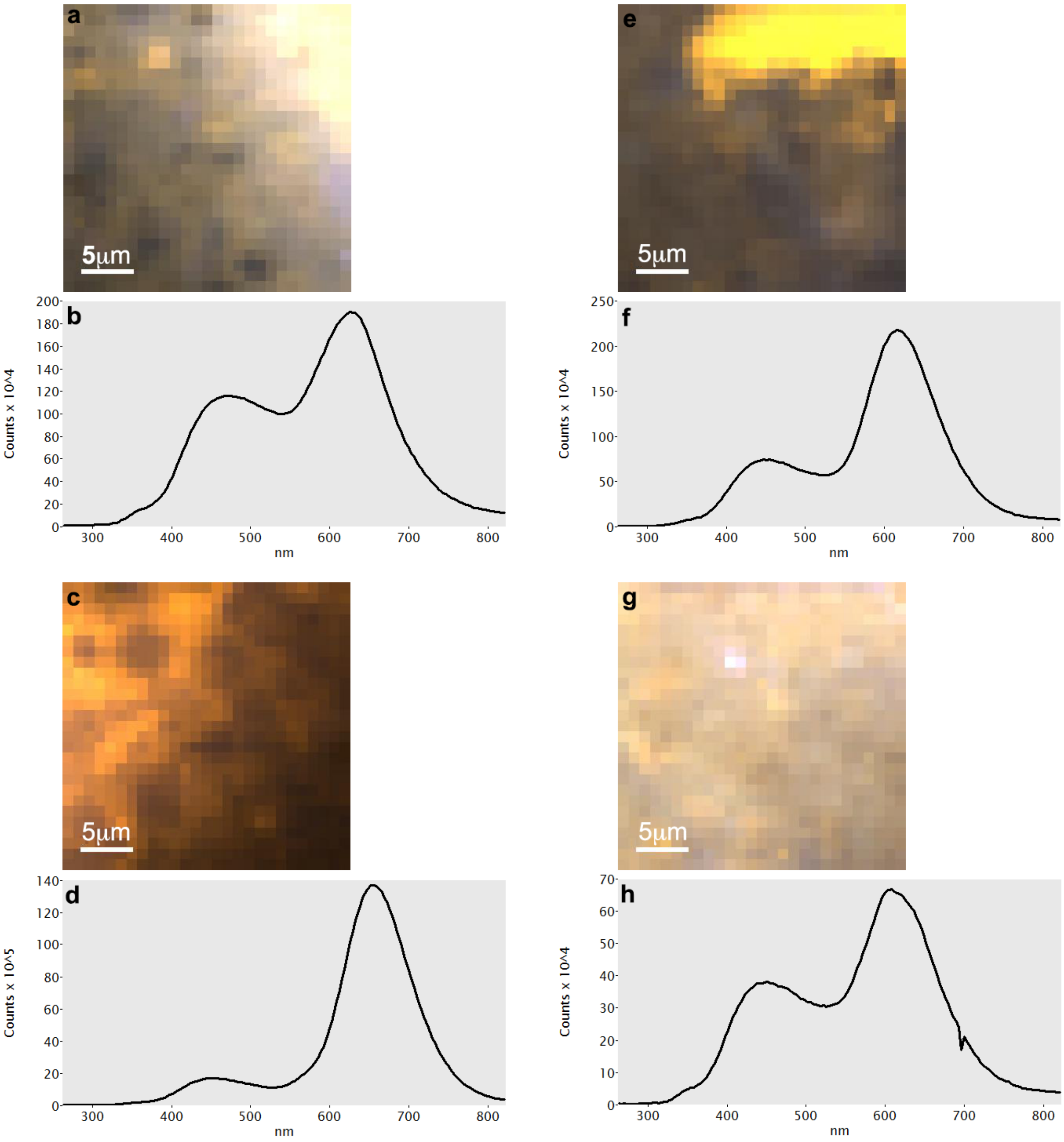

Supplementary Figure S4. Spectrum imaging maps and SEM-CL spectra of standard reference samples. (**a**, **b**) Chalk 2. (**c**, **d**) Limestone 2. (**e**, **f**) Marble 2. (**g**, **h**) Marble 3.

Supplementary Figure S5

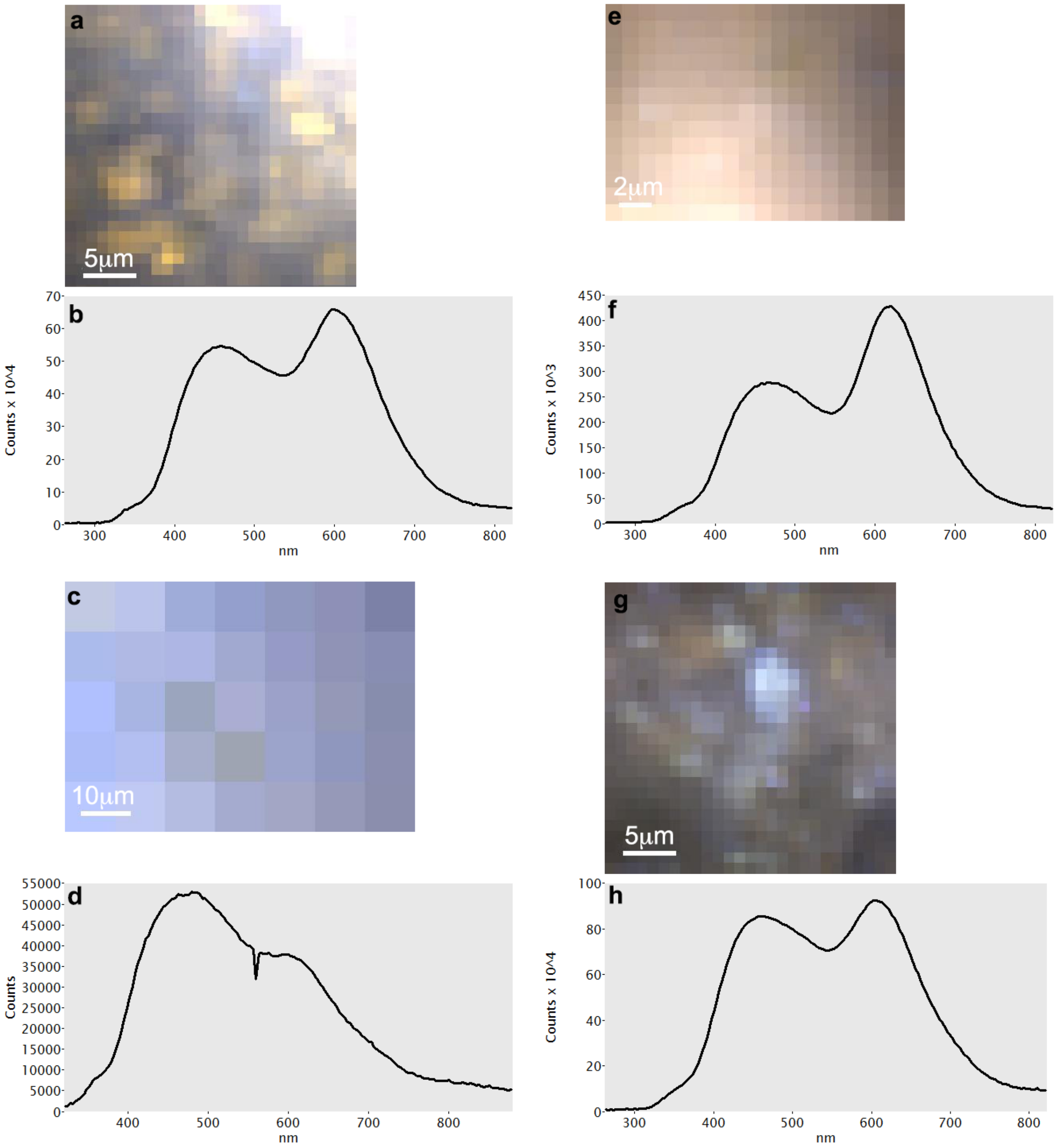

Supplementary Figure S5. Spectrum imaging maps and SEM-CL spectra of standard reference samples. (**a**, **b**) Nari. (**c**, **d**) Plaster C1. (**e**, **f**) Plaster C3. (**g**, **h**) Plaster C4.

Supplementary Figure S6

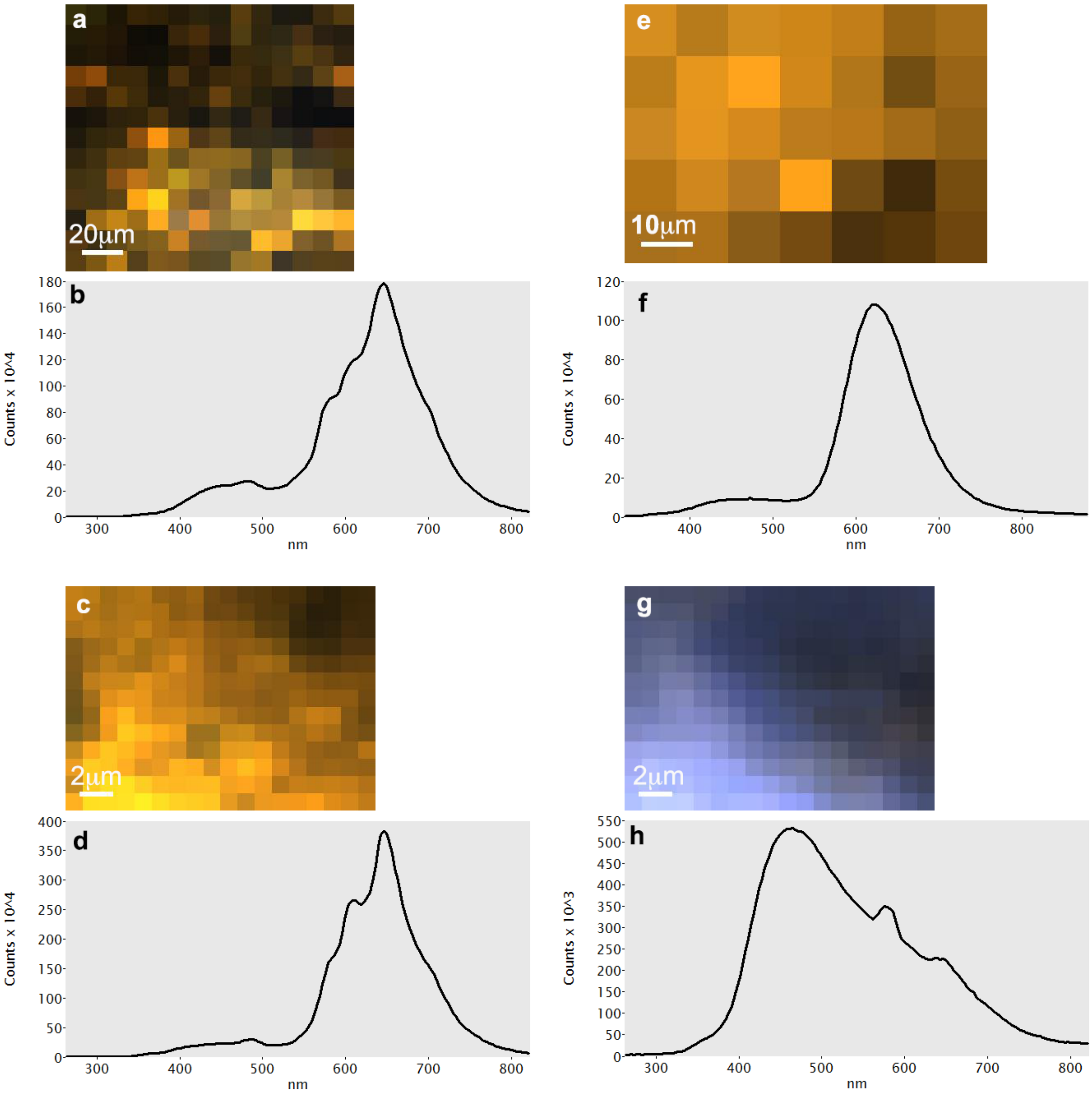

Supplementary Figure S6. Spectrum imaging maps and SEM-CL spectra of standard reference samples. (**a**, **b**) Aragonite 1. (**c**, **d**) Aragonite 3. (**e**, **f**) Aragonite 4. (**g**, **h**) Aragonite 5.

Supplementary Figure S7

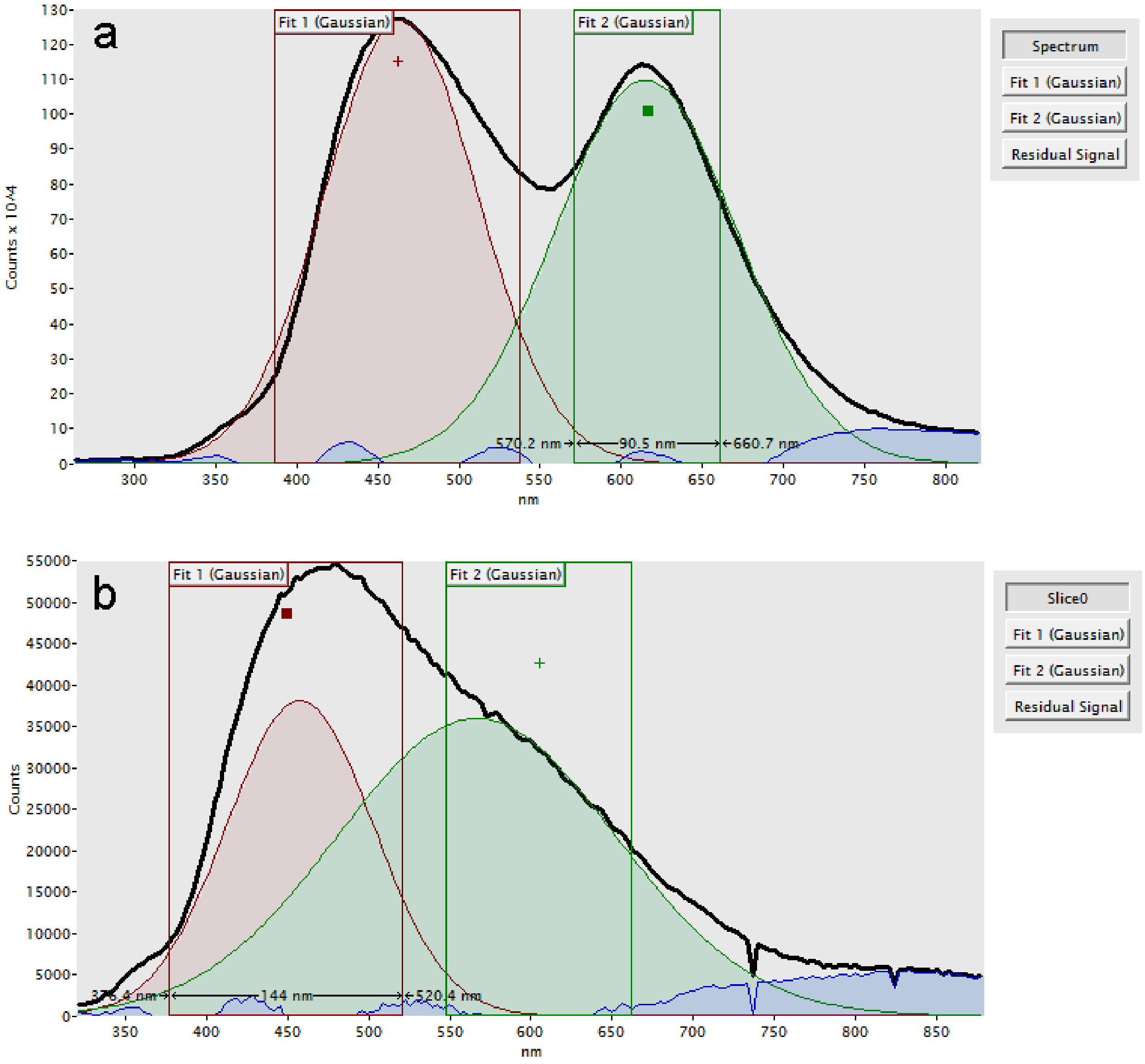

Supplementary Figure S7. Peak fitting of SEM-CL spectra. (**a**) Spectrum showing blue and orange emissions. (**b**) Spectrum showing dominant blue emission that masks a shoulder in the range of yellow.

Supplementary Figure S8

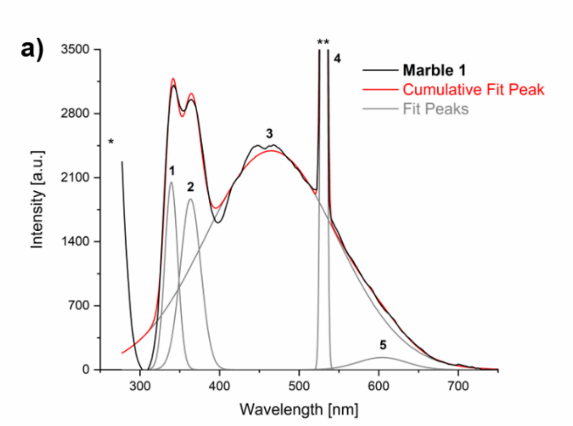

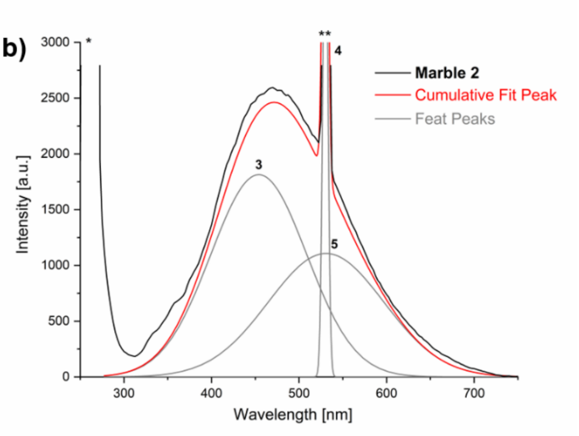

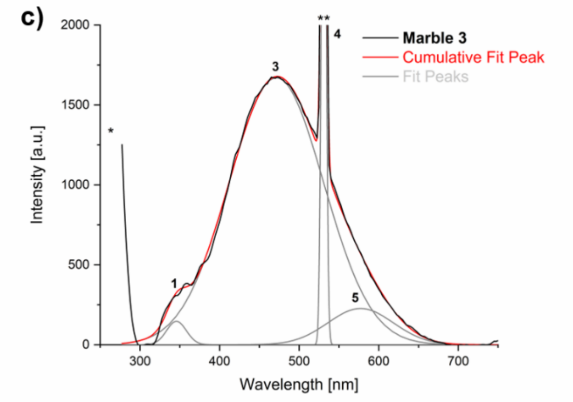

d)

Sample	Peak 1	Peak 2	Peak 3	Peak 4	Peak 5
	nm				
**Marble 1**	340	364	464	531	604
**Marble 2**	-	-	454	531	531
**Marble 3**	346	-	471	531	577

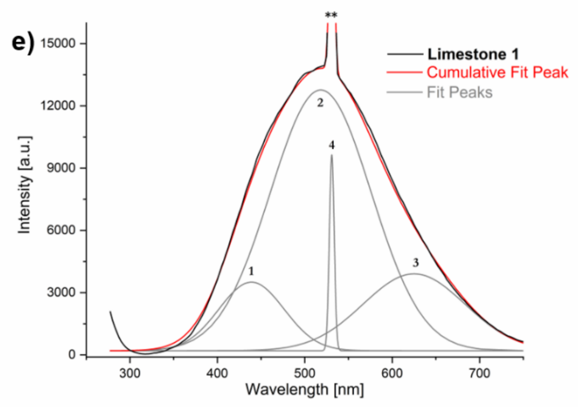

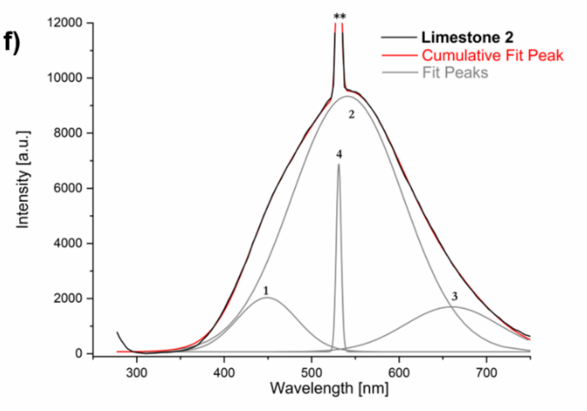

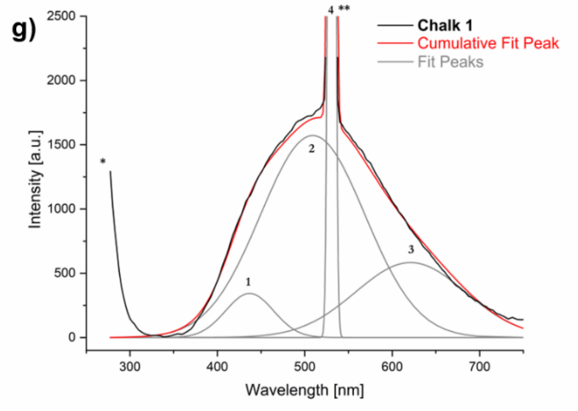

h)

Sample	Peak 1	Peak 2	Peak 3	Peak 4
	nm			
**Limestone 1**	439	518	625	531
**Limestone 2**	449	541	660	531
**Chalk 1**	436	509	620	531

Supplementary Figure S8. Peak fitting of LIF spectra of marble, limestone and chalk samples.

Supplementary Figure S9

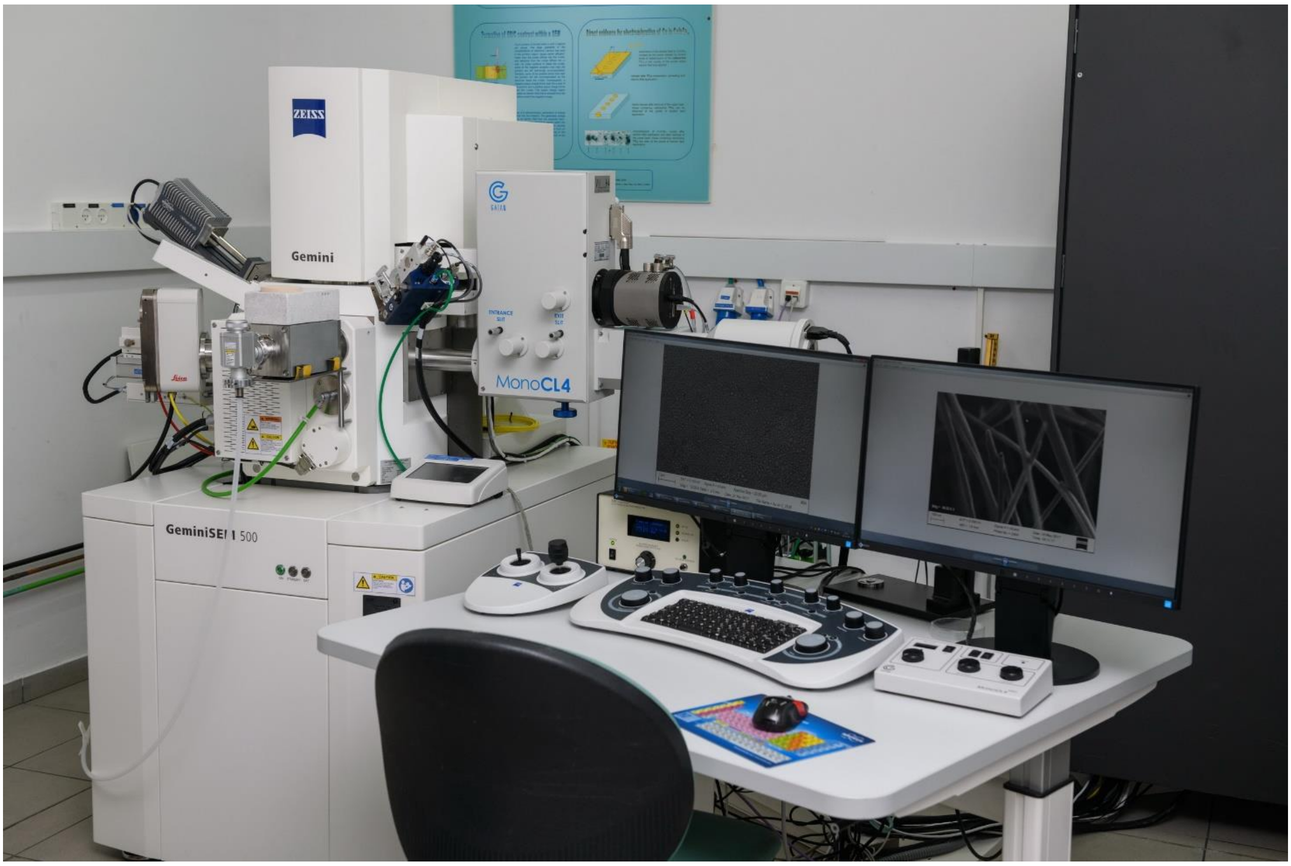

Supplementary Figure S9. SEM-CL analytical setup at the Moskowitz Center for Nano and Nano-Bio Imaging, Weizmann Institute of Science.

Supplementary Figure S10

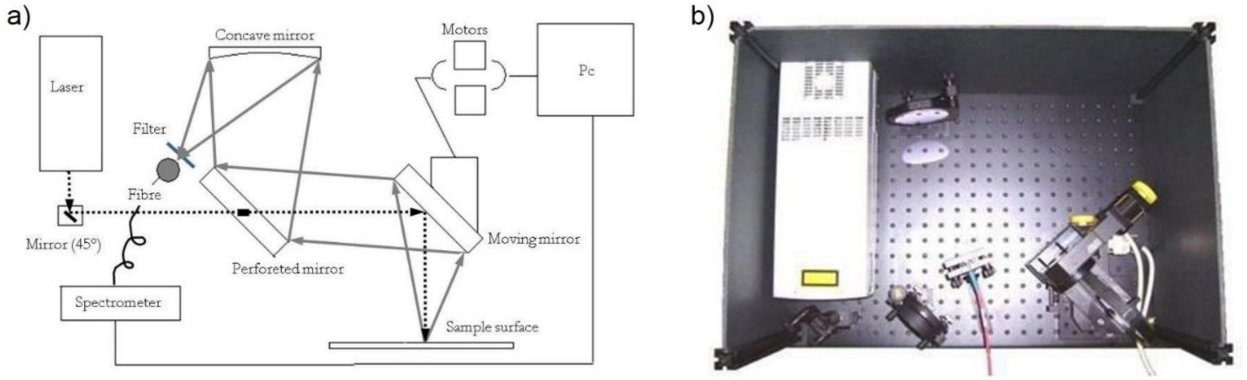

Supplementary Figure S10. LIF analytical setup at the Technical Unit for the Development of Applications of Radiations, ENEA. (**a**) Schematic representation of the apparatus. (**b**) Top view of the instrument.
